# A recursive enzymatic competition network capable of multitask molecular information processing

**DOI:** 10.1038/s41557-025-01981-y

**Published:** 2025-10-17

**Authors:** Souvik Ghosh, Mathieu G. Baltussen, Anna C. Knox, Rianne Haije, Quentin Duez, Anastasia T. Tsitsimeli, Man Him Chak, Jonathon E. Beves, Wilhelm T. S. Huck

**Affiliations:** 1 Institute for Molecules and Materials, Radboud University, Nijmegen, Netherlands; 2 School of Chemistry, UNSW Sydney, Sydney, New South Wales Australia

**Keywords:** Cheminformatics, Supramolecular chemistry

## Abstract

Living cells understand their environment by combining, integrating and interpreting chemical and physical stimuli. Despite considerable advances in the design of enzymatic reaction networks that mimic hallmarks of living systems, these approaches lack the complexity to fully capture biological information processing. Here we introduce a scalable approach to design complex enzymatic reaction networks capable of reservoir computation based on recursive competition of substrates. This protease-based network can perform a broad range of classification tasks based on peptide and physicochemical inputs and can simultaneously perform an extensive set of discrete and continuous information processing tasks. The enzymatic reservoir can act as a temperature sensor from 25 °C to 55 °C with 1.3 °C accuracy, and performs decision-making, activation and tuning tasks common to neurological systems. We show a possible route to temporal information processing and a direct interface with optical systems by demonstrating the extension of the network to incorporate sensitivity to light pulses. Our results show a class of competition-based molecular systems capable of increasingly powerful information-processing tasks.

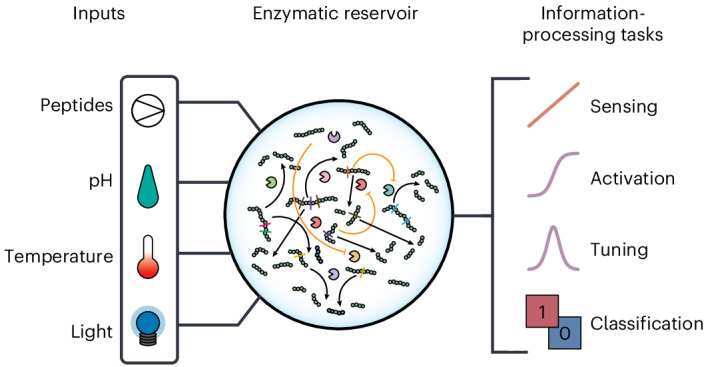

## Abstract (web summary)

Designing enzymatic reaction networks capable of mimicking the complexity of biological information processing is challenging. Now, an in chemico reservoir sensor based on a recursive enzymatic competition network has been designed that can process diverse physical and chemical inputs and perform several information-processing tasks.

## Main

Living systems sense, process and classify information from their environment using complex reaction networks^1,2^. These networks can respond to diverse physical and chemical stimuli such as the availability of nutrients, pH levels, changes in temperature and variations in light levels^3^. Seminal work has shown that complex networks found in nature share structural design patterns^4^. These so-called ‘network motifs’ have served as a template for a wide range of synthetic reaction networks^5^ that exhibit well-defined behaviour, such as spatiotemporal dynamics^6–9^, robustness and adaptation^10–12^, and computational capabilities^13–18^. Importantly, none of the experimental approaches to functional enzymatic reaction networks reported to date fully capture the complexity of living systems. The focus on bottom-up construction of small network motifs is hampered by increasingly complex and laborious molecular designs. To emulate the rich information-processing behaviour found in cells, a new, scalable approach to artificial enzymatic reaction networks is needed.

Recently, we have shown that a self-organizing reaction network based on the prebiotic formose reaction forms a powerful chemical reservoir computer capable of complex systems predictions and forecasting^19^. Small biochemical networks are unsuitable for such in chemico reservoir computation as these are designed for very specific tasks and operate within narrowly defined parameter space^20^. In contrast, reaction networks with recursive interactions (such as the prebiotic formose reaction) may generate a wide array of chemical products that are non-linearly dependent on a small number of inputs.

Inspired by the adaptive, multifaceted information processing found in cells, we present a blueprint for scalable, enzymatic reservoir computing. By combining resource competition with recursive interactions, a complex, non-linear enzymatic reaction network emerges in response to changes in reaction conditions, without the need for a fixed network motif. Theoretical studies have shown how crosstalk and resource competition endow highly interconnected protein interaction networks with computational capabilities^21–23^, and recent research has shown competition may play a role in neuronal processes^24^.

In this study, we experimentally demonstrate that recursive enzymatic competition networks can process physicochemical information in the environment by acting as a reservoir sensor. We designed a protease-based enzymatic reaction network which receives up to seven short peptide substrates as input. Each of the peptides contains multiple cleavage sites for different proteases with different reaction rates. The many possible cleavage pathways lead to competitive and recursive interactions, yielding a highly non-linear network of enzymatic reactions that can be used for various sensing tasks. We first show the capacity of this network for reservoir computation by performing several non-linear classification tasks of different substrate input compositions. Next, we extend the physical reservoir paradigm to physicochemical sensing tasks by incorporating temperature and pH levels as new inputs. We demonstrate the potential of the enzymatic reservoir for general in chemico information processing by the simultaneous sensing, non-linear processing, and classification of changes in its environment. The reservoir can differentiate temperatures reliably with an average error of 1.3 °C over a broad range of temperatures (25–55 °C), and can accurately perform activation, tuning and decision-making tasks over that same range. Finally, we extend the network to detect changes in light-pulse periodicity for the range between 2 and 8 min. Our work combines principles from cellular reaction networks and neuromorphic computing into a novel paradigm to develop physicochemical sensors suitable for tasks in a biological environment, a next step towards intelligent autonomous molecular systems.

## Results and discussion

### Design of a recursive enzymatic competition network

**Fig. 1:**
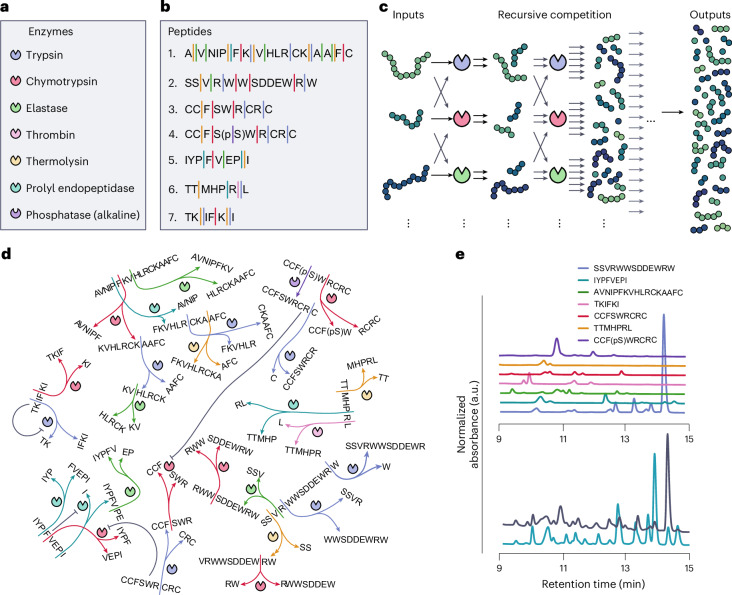
Design of a recursive enzymatic competition network. **a**,**b**, Lists of enzymes (**a**) and substrates (**b**) used in the reservoir design. Colour-coded vertical bars indicate potential cleavage sites for corresponding enzymes. **c**, Schematic representation of the competitive and recursive nature of enzyme–substrate interactions orchestrated within the reservoir, leading to a complex mixture of peptide fragments. **d**, Schematic overview of enzymatic interactions in the designed recursive ERN. Not all possible cleavage pathways are shown. **e**, Top: Chromatograms showing cleavage patterns in the network for individual peptides. Bottom: comparison between the accumulated cleavage patterns of individual peptides (grey) and the cleavage pattern resulting from using all peptides as input simultaneously (blue). Source data

The design of our enzymatic reservoir is based on a new class of scalable enzymatic reaction networks that are connected through shared substrates and the recursive use of reaction products. We selected seven enzymes (listed in Fig. 1a): six proteases which selectively cleave peptides at different amino acid residues (trypsin, chymotrypsin, elastase, thrombin, thermolysin and prolyl endopeptidase) and alkaline phosphatase (Supplementary Table 3). We then selected seven short peptides, each containing multiple cleavage sites for two or more enzymes (listed in Fig. 1b). The cleavage fragments can serve as substrates for further cleavage (Fig. 1c). The multitude of cleavage reactions leads to competition between the peptides for available enzymes. Furthermore, the peptides CCFSWRCRC (**3**), IYPFVEPI (**5**) and TKIFKI (**7**) serve as slow substrates for chymotrypsin, prolyl endopeptidase and trypsin, respectively^25,26^. Such substrates act as effective reversible inhibitors that are slowly degraded, yielding non-linear kinetics that resemble autoactivation. The peptide library also includes a reversible peptide proinhibitor of chymotrypsin which features a phosphorylated serine residue (CCF(pS)WRCRC, **4**). This phosphate group can be dephosphorylated by alkaline phosphatase, restoring its inhibitory effect on chymotrypsin and introducing competition with other substrates. The repeated cleavage of peptides by the enzymes leads to the formation of a recursive enzymatic reaction network that is characterized by resource competition for catalysts and emergent inhibitory feedback mechanisms (a schematic of possible cleavage pathways is shown in Fig. 1d), and can generate complex mixtures of output fragments. The actual reaction network varies based on the specific competing interactions between the enzymes and substrates at any particular combination of input concentrations and physicochemical conditions.

We immobilized the enzymes onto monodisperse polyacrylamide hydrogel beads, using a previously described approach^27^. Immobilization of enzymes allows us to separate the enzymatic ‘hardware’ from the peptide substrates ‘software’ by compartmentalizing the enzymatic reaction network (ERN) inside a continuous stirred tank reactor (CSTR), effectively making the enzymatic reservoir reusable (see Methods for details). Prior to constructing the full network in the CSTR, we tested the activity of each individual enzyme (Supplementary Fig. 1) and determined peptide concentration ranges that gave a substantial response in output. We further examined the cleavage fingerprint of individual peptides produced by the full set of immobilized enzymes using high-performance liquid chromatography (HPLC) (Fig. 1e, top; see Supplementary Information, section 3.2.2 for details), and the fingerprint obtained when introducing all peptides simultaneously (Fig. 1e, bottom). The clear difference in output produced establishes that the competition resulting from the introduction of multiple peptides leads to a significant change in molecular interactions.

### Chemical and physicochemical non-linear classification tasks

**Fig. 2:**
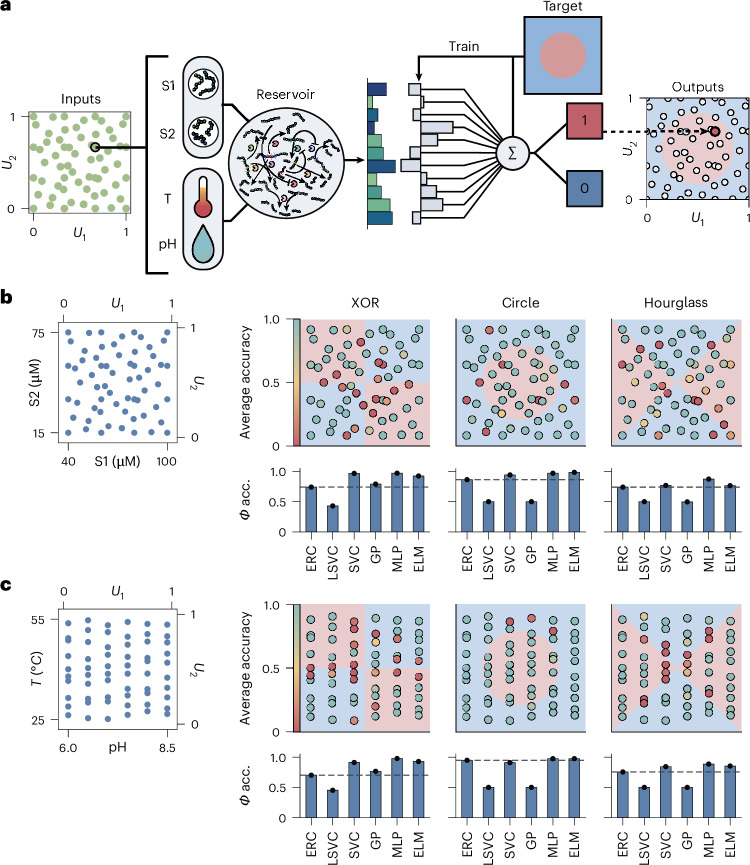
Chemical and physicochemical non-linear classification tasks. **a**, Schematic of the procedure used to classify input conditions. Every input condition (varying parameters *U*_1_ and *U*_2_) corresponds to a chemical output composition, which is multiplied by pretrained weights based on a target classification function. The result is summed to a final computational output, resulting in the classification of the input. **b**, Non-linear classification based on two different sets of substrate inputs (S1 and S2). On the left side is shown the input space. Next are shown the results of classification for an XOR gate, circle classification and hourglass classification, with the background indicating the desired classification output (blue = 0, red = 1). Locations of the markers indicate the corresponding location in input space, with the colour indicating the average test-set accuracy of that input point for 130 different L5O train-test splits, with +1 corresponding to perfect predictions, and 0 to total failure (Methods). The bar charts below every classification result show the average L5O-CV *Φ* accuracy for the enzymatic reservoir (ERC), the training algorithm without reservoir (LSVC), and several machine-learning algorithms (SVC, support vector classifier; GP, Gaussian process; MLP, multilayer perceptron; ELM, extreme learning machine). **c**, Non-linear classification results using pH and temperature as inputs, where similar to **b** the input space is shown on the left, and the respective accuracies per input point and a comparison of average accuracy to different in silico algorithms are shown on the right. Source data

First, we demonstrate that our ERN has sufficient non-linear dynamics to exhibit reservoir computing capabilities. We assembled the ERN in a CSTR and distributed the peptides into input syringes based on their effective concentration regimes. We combined peptides **1** and **2** in syringe S1, peptides **3**–**5** in syringe S2, and peptides **6** and **7** were distributed in both S1 and S2. Syringes S1 and S2 served as potential input variables, with input concentrations (effective concentration in the reactor) varying from 40 to 100 μM for S1 and from 15 to 75 μM for S2. The concentrations of peptides **6** and **7** were kept constant at an input concentration of 160 μM, and the total flow rate of the system was kept constant by compensating the change in flow rate of S1 and S2 with an opposite change in flow rate of a buffer solution. The concentration ranges were specifically selected based on experimental observability, solubility, detectable cleaved fragments and non-linear effects (see Supplementary Information, sections 3.2.1 and 3.2.2 and Supplementary Fig. 4 for more details). To increase the experimental throughput of our system, we coupled the reactor directly to an electrospray ionization mass spectrometer (ESI-MS) (see Supplementary Fig. 3 for an overall schematic of the set-up). For each input combination, the network was allowed to reach steady-state over a 1-h equilibration period, during which 103 different ion traces resulting from specific cleaved fragments were recorded. The final output corresponding to each input was obtained as the averaged ion intensities over the final 10 min of the equilibration period (Methods and Supplementary Fig. 17). The output of the ERN was converted to a ‘computational’ output by training a single linear readout layer (a standard approach in physical reservoir computing), which multiplies every output feature with a weight and sums the resulting weighted signals (Fig. 2a). For the classification tasks, we applied a linear support vector classifier (LSVC) algorithm to obtain the correct weights, resulting in a binary classification for every input.

We first performed the XOR, circle and hourglass classification tasks using S1 and S2 as inputs, randomly sampling the input space in an appropriate range of input concentrations (Fig. 2b). For every input, the reservoir output was collected and trained as described above. To validate the performance of the classification tasks, we calculated the average *Φ* accuracy (also known as the phi coefficient, or Matthews correlation coefficient) using a repeated stratified leave-5-out cross-validation (L5O-CV) following Baltussen et al.^19^, and we compared these results to standard in silico classification methods. The results shown in Fig. 2b demonstrate that the ERN reservoir can perform these classification tasks on peptide inputs with a similar accuracy as established machine-learning techniques performing classification on an equivalent input space.

To further verify that the full set of enzymes is necessary to perform these classification tasks, we tested the performance of these classification tasks for subsets of the system with respectively three and five enzymes, instead of the full set of seven enzymes (Supplementary Fig. 15). The inability to perform non-linear classifications with these smaller networks demonstrates that the computational capacity is not straightforward, but an emergent property of the constructed network. We also investigated how many different output species need to be generated or observed to obtain sufficient performance. Depending on the task, anywhere from fewer than 50 to the full dataset of 103 different peptide traces are required to achieve optimal performance (Supplementary Fig. 16). To evaluate the ability of the reservoir for time-series prediction, we applied a dynamic input based on a sine wave. Output signals were sampled every 4.5 min over 7 h. A ridge regression model was trained to predict the input signal at future time points (9 and 13.5 min ahead). The results of the prediction are shown in Supplementary Fig. 12, demonstrating that the ERN reservoir is indeed capable of short-term forecasting (see Supplementary Information, section 3.2.5 for full details).

Having established the reservoir computing capabilities of the network based on peptide inputs, we wished to explore the capacity of our ERN to classify inputs from the physical environment in the form of temperature and pH, demonstrating that a change in peptide concentration is not necessary for the network to exhibit reservoir capabilities. The activity of each enzyme in the network will have a characteristic dependence on temperature and pH. Changes in these parameters therefore alter the relative strengths of connections in the network and lead to an observable response in the reservoir output (Supplementary Figs. 5–8). To perform purely physicochemical classification, we restrict the input space to only changes in temperature and pH, maintaining a constant input concentration of all peptides (Fig. 2c). Similar to the S1–S2 inputs described above, we recorded 60 steady-state outputs by randomly sampling 10 different temperature inputs within the range of 25–55 °C in combination with six different pH values ranging from 6 to 8.5 (controlled by separate buffer-syringes, Methods). Figure 2c shows the resulting L5O-CV *Φ* accuracies for three tasks (XOR, circle,and hourglass classification) of the ERN in comparison to in silico techniques on a similar input space. Comparable results for peptide–temperature and peptide–pH input combinations are shown in Supplementary Fig. 9. Clearly, changes in physicochemical parameters are sufficient inputs for the network to perform reliable classification of its environment. To demonstrate the broad application to non-linear classification tasks, we provide an extended set of tasks in Supplementary Fig. 14.

### Generalized physical information processing

**Fig. 3:**
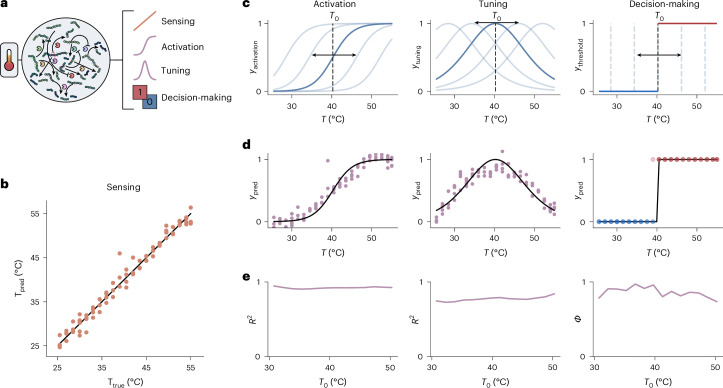
Generalized information processing of temperature. **a**, Schematic overview showing the classes of information-processing tasks the enzymatic reservoir can perform using temperature as an input. **b**, Plot showing the true versus predicted temperature. The datapoints shown are obtained from separate L5O validation runs (only the test set is shown). The average s.d. over all validation runs is 1.33 °C. **c**, Plots showing example activation, tuning and decision-making tasks at different inflection/tuning/classification boundaries *T*_0_, with *T*_0_ = 40 °C emphasized by the dashed vertical line (double-headed arrows indicate that boundaries can be shifted left or right). **d**, Plots showing predictions over the full range of temperature inputs, obtained from multiple L5O validation runs for a specific version of each task class, with *T*_0_ = 40 °C. Every point represents a test-set prediction for a different trained readout layer. The black line indicates the true output of the task. **e**, Plots showing the average L5O test-set *R*^2^ and *Φ* scores over the full range of tasks, with the *x* axis representing the inflection point, tuning point or classification boundary *T*_0_. Source data

Information-processing networks in cells are not generally limited to single tasks but demonstrate adaptive responses over a broad range of possible input conditions that may then be picked up by subsequent networks for further fine-tuning of cellular behaviour^28,29^. We demonstrate the capacity of the reservoir for four unique classes of information-processing tasks: linear sensing, activation, tuning and finally decision-making, in a generalized fashion, using temperature as input, without requiring further modification of the ERN (Fig. 3a and Methods). Specifically, the sensing task requires the network to estimate the environmental temperature based on its output composition, which depends on the reservoir producing a linear response over the full range of temperatures. The activation task corresponds to estimating a sigmoidal response as a function of temperature, a function commonly encountered in both biological and machine-learning applications^30^, and requiring a non-linear response of the reservoir around the infliction point *T*_0_ and a static response towards the asymptotes. The tuning task corresponds to estimating the gradient of the activation task, requiring a local pulse-like response around *T*_0_, an important feature for learning, and similar to neuronal activity patterns found in the brain^31^. Finally the decision-making task was implemented as a binary classification task, requiring a switch-like response of the reservoir at the classification boundary *T*_0_. All tasks thus require different sets of behaviours that can consistently be decoded by a linear readout layer. In the case of the continuous tasks (sensing, activation, tuning) a ridge regressor was used to train the linear readout layer. For the decision-making task, similar to the previously described classification tasks, a LSVC was used. The reservoir was tested over a large range of temperature conditions (25.5–55 °C) at 1.5 °C intervals at fixed pH and peptide inputs, with outputs obtained as quadruplicates. Specifically for the activation, tuning and decision-making tasks the capacity of the reservoir to correctly estimate a large range of different inflection points, tuning points and classification boundaries was evaluated (examples shown in Fig. 3c).

As shown in Fig. 3b, the reservoir is capable of sensing temperature across the full range of tested conditions (a 30 °C interval) with an average s.d. of 1.33 °C, obtained via multiple L5O tests. Moreover, the same broad performance is observed for the activation, tuning and decision-making tasks, with the reservoir capable of making accurate predictions across the full range of temperatures. The network achieves slightly lower scores for the tuning task compared with the activation task. This functionality is more challenging to perform because the network must account for twice the amount of non-linearity compared with the activation task, while still relying on only a linear readout. The reliable performance of the ERC across all classes of tasks shows that the recursive network creates both linear and various non-linear responses as a function of temperature, which can subsequently be extracted using only a linear readout layer.

### Extending the input space to light pulses

**Fig. 4:**
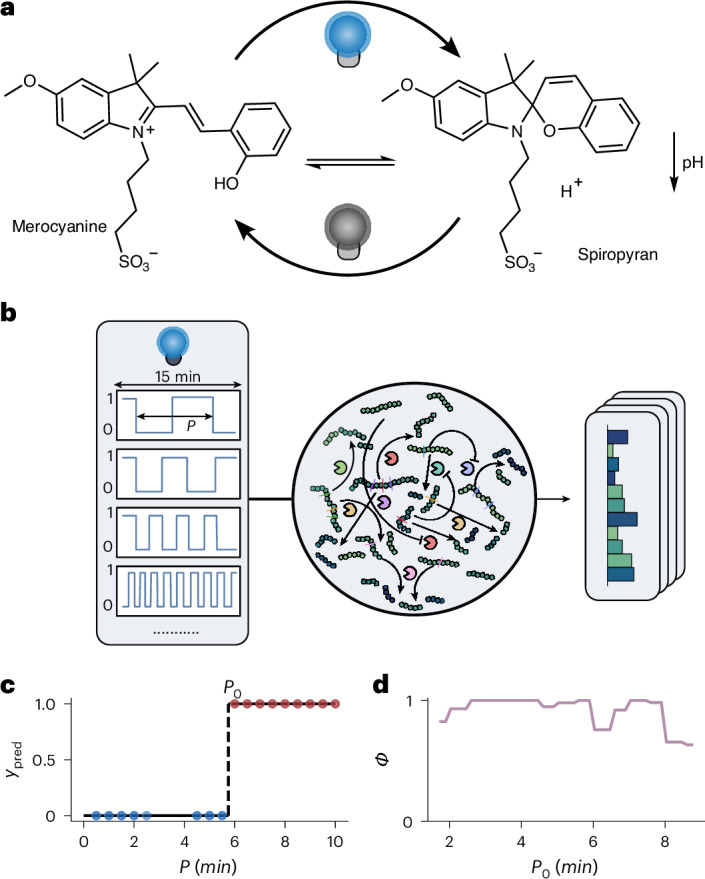
Extending the network to light-pulse sensitivity. **a**, Blue light irradiation triggers merocyanine-to-spiropyran switching, leading to a decrease in pH. **b**, Schematic experimental overview for the light frequency sensor. **c**,**d**, Plots showing a binary switching task (**c**) and *Φ* accuracies of the binary switching task (**d**) as a function of light-pulse periodicity thresholds (*P*_0_). Red and blue dots in **c** indicate data points on different sides of the decision boundary. Source data

Finally, as a first proof-of-principle of the extensibility of our enzymatic reservoir sensor towards detecting patterns in light intensity and direct interfacing with optical devices, we demonstrate the detection of the periodicity of a periodic blue light pulse. To achieve this task, we extend the reservoir by the inclusion of a merocyanine dye in the buffer solution (Fig. 4a), a photochromic compound that can decrease the solution pH by 3.5 units upon irradiation with blue light^32^. As we have shown above that the reservoir is sensitive to changes in pH, we expect the reservoir to respond to changes in light upon addition of this dye (Supplementary Fig. 11). Following a procedure similar to the temperature switch introduced above, we maintained a fixed temperature (30 °C) and substrate input concentrations, and exposed the reactor to 17 different light-pulse periodicities (from 30 s to 600 s) with constant average intensity for 15 min (Fig. 4b). The network outputs were measured offline using HPLC. Importantly, the measured data reflect the cumulative dynamic response of the enzymatic reservoir induced by the light pulses with different periodicities. We then perform a binary classification with respect to a threshold periodicity *P*_0_, establishing a light-sensitive switch over a broad range of threshold periodicities (Fig. 4c). We find that the reservoir accurately differentiates timescales across a range of periodicities between 2 and 8 min (Fig. 4d). These results are remarkable as the network is apparently sensitive to time-dependent changes in pH while the average pH in the reactor is constant. This experiment demonstrates the potential of the enzymatic reservoir to detect temporal information in the environment, offering opportunities for interfacing the enzymatic reservoir with information generated by electronic devices.

## Conclusions

We have introduced a new scalable approach to the design of complex enzymatic reaction networks with emergent behaviour, based on recursive competition for resources. We have shown that this network has in chemico reservoir computation capabilities, can be used as an enzymatic sensor to detect physicochemical changes in its environment and is scalable to new physical inputs. The network is capable of a diverse set of information-processing tasks, showing strong performance on linear sensing, non-linear processing and classification based on both chemical and physical inputs.

We have further showcased how the computational capacity scales up with network complexity by varying the number of enzymes or taking into account smaller subsets of output features. Our approach is inherently scalable: additional enzymes (other proteases, phosphatases or kinases) can be readily added along with additional or longer peptides that contain multiple cleavage or (de)phosphorylation sites. One can also envisage activating or deactivating peptides as substrates for the proteases by enzymatic (de)glycosylation. We therefore expect that the computational performance of the enzymatic reservoir sensor can be further improved. The development of enzymatic reservoirs could be further aided by in silico surrogate models. These could be used to efficiently map and search the high-dimensional space of chemical and physical parameters in which the network would perform optimally for a specific set of computational tasks.

We have demonstrated that the network’s response can be modulated by physical stimuli such as pH, temperature and light. This tunability opens up promising avenues for engineering dynamic responses from the system by expanding the range of input parameters. One could, for instance, incorporate enzymes or enzyme cascades that modulate the local pH environment in response to an analyte of interest (for example, ATP/ADP ratio, or the presence of certain sugars in a mixture), thereby influencing the reservoir response.

The enzymatic network reported here is the second example of a chemical reaction network capable of reservoir computing. This network shows somewhat inferior computational capabilities compared to the previously reported formose reservoir, which outperformed several in silico machine-learning algorithms in non-linear classification tasks and performed particularly well on dynamic tasks. To further increase the computation capabilities of the enzymatic network, future work could focus on adding additional peptides and proteases to deepen the complexity of the system. An expansion of the number of peptides used could also offer routes to task-adaptive fine-tuning of the responsiveness of the enzymatic reservoir. Comparing the two chemical systems, the formose reaction operates under rather harsh reaction conditions, not compatible with many other chemical systems. In contrast, the reaction conditions of the enzymatic reservoir are mild, and we anticipate that the enzymes could be embedded in hydrogels or other soft materials, allowing for a direct interface between in chemico reservoir computing and responsive materials.

Potentially, a direct cellular interface may be envisioned by replacing the mass spectrometry-based readout layer with an in vitro variant of biochemical decision-making networks that have been shown to function in living cells, such as a metabolic perceptron^33^ or a protein-based winner-takes-all network^34^. These biochemical networks would replace our in silico regression models, and their output could then be trained to influence cellular behaviour, thus creating a link between the information-processing capabilities of the reservoir and living systems.

Finally, the capacity for enzymatic reaction networks to process time-based information and the sensitivity to changes in the periodicity of light signals, opens a wide range of possibilities for dynamic sensing and encoding of temporal information in chemical systems using optical pulses. Optically interfacing electronic computers with chemical systems represents a major step towards autonomous molecular systems.

## Methods

### Materials

All chemicals and reagents were used as received from commercial suppliers without any further treatment unless stated otherwise. Enzymes were purchased from Merck Sigma-Aldrich. Specifications are listed in Supplementary Information, section 1.1.1. All enzymes are assumed to be salt-free for calculating enzyme concentration, and calculations are based on specified molecular weights.

All peptides used in this study were synthesised by CASLO as trifluoroacetate salts with >95% purity. Peptide stock solutions (10 mM) were prepared in dimethylsulfoxide and kept at −20 °C. Product numbers are listed in the Supplementary Table 1.

General experiments were performed using 50 mM phosphate buffer, pH 7.4. Experiments requiring different pH conditions were performed using Britton–Robinson buffer (pH). The phosphate buffer was prepared as follows: potassium phosphate dibasic (5.352 g) and potassium phosphate monobasic (1.475 g) were mixed in MilliQ water to total volume of 1,000 ml, yielding 50 mM of final concentration. The mixture is adjusted to pH 7.4 solutions by addition of 2 M HCl and 10 M NaOH. The change in volume due to the addition of 10 M NaOH and 2 M HCl was neglected.

### Flow reactions

A poly(methyl methacrylate) CSTR (volume, 100 μl) was prepared as described in Baltussen et al.^27^ For reactions involving temperature control, in place of a poly(methyl methacrylate) plate, the reactor was constructed above an aluminium plate such that the plate comprised the floor of the reactor. An image of the reactor set-up is available in Supplementary Fig. 3. This enabled high-precision control and monitoring of the temperature inside the reactor. Enzyme beads (4 µl each; total, 28 µl) were fixed inside the CSTR with a polycarbonate membrane of 10-µm pore size over the inlet and outlet. A LabM8 syringe pump platform was used to control input flow rates and temperature. Syringes were prepared with peptide solutions with each concentration configured to enable a combined flow rate of 400 μl h^−1^.

### Chemical inputs

The peptides listed in Fig. 1b were used as chemical inputs/substrates for enzymes. Peptides **1** and **2** were combined in syringe S1, while peptides **3,** **4** and **5** were combined in syringe S2. Peptides **6** and **7** were distributed across both syringes. Peptides in syringe S1 and S2 served as potential input variables, with input concentrations (effective concentration in the reactor) varying from 40 to 100 μM for S1 and from 15 to 75 μM for S2. The concentration of peptides **6** and **7** was kept constant at an input concentration of 160 μM for all the experiments. The total flow rate of the system was kept constant by compensating the change in flow rate of S1 and S2 with an opposite change in flow rate of a buffer solution. The total flow rate was maintained at 400 µl h^−1^ for most experiments, except for the light-pulse experiments, where a flow rate of 800 µl h^−1^ was used. For the S2 versus *T* and S2 versus pH classifications, the concentration of peptides (**1** and **2**) in S1 were kept constant at 70 µM, whereas the peptides (**3**, **4** and **5**) in S2 were randomly varied between 15 and 75 µM, with respective pH and temperature conditions. For the rest of the experiments, peptide concentrations were held constant: peptides **1** and **2** were maintained at 70 µM, peptides **3**, **4** and **5** at 45 µM, and peptides **6** and **7** at 160 µM each. For each input combination, the network was allowed to reach steady state over a 1-h equilibration period. To increase the experimental throughput of our system, we coupled the reactor directly to a trapped ion-mobility time-of-flight (TIMS-ToF) mass spectrometer. The final output of the reservoir was obtained as the averaged ion intensities over the final 10-min sample period of the steady-state output (Supplementary Fig. 17).

### Physicochemical inputs

#### Temperature

The reactor temperature was controlled via a custom, tuned heating unit in the aluminium plate compromising the base of the CSTR. This system featured a 50-W heater cartridge with a PT100 temperature sensor. This heating unit was controlled by the same central unit as the flow rate. For S2 versus *T* and pH versus *T* classifications, temperatures between 25 °C and 55 °C were randomly sampled with changing peptide concentration. Finally, for generalized physical information processing, a large range of temperature conditions (25.5–55 °C) at 1.5 °C intervals were tested.

#### pH

Britton–Robinson buffers of pH 6.0, 6.5, 7.0, 7.5, 8.0 and 8.5 were prepared as follows: boric acid (3.0915 g), 85% aqueous orthophosphoric acid (3.42 ml) and glacial acetic acid (2.86 ml) were mixed and diluted to total volume of 1,000 ml, yielding 50 mM of every acid in the solution. The mixture is adjusted to the respective pH solutions by addition of 2 M HCl and 10 M NaOH. The change in volume due to the addition of 10 M NaOH and 2 M HCl was neglected.

For S2 versus pH and pH versus *T* classifications, the above-specified pH values were varied with changing peptide concentration and temperature.

#### Light

Light-emitting diode lights were purchased from Avonec (5 W, *λ*_max_ = 460 nm; 3 W) and powered by a Thorlabs LEDD1B driver. The timing and the intensity of this light source were externally controlled by an Arduino device. Blue light pulses were applied in alternating on/off cycles of 30–600 s for a total duration of 15 min. A 30-s pulse induced a significant pH drop, while the longest cycles allowed the pH to return closer to its initial value.

### Mass spectrometry

The output of the reactor was measured in real-time using a TIMS-ToF mass spectrometer (Bruker Daltonics). Once the CSTR was filled, the output of the reactor was coupled to an inline check valve, a dilution line (providing 0.046 ml min^−1^ of MilliQ water) and a back-pressure regulator as specified in Baltussen et al. prior to connection to the TIMS-ToF mass spectrometer^19^. The outflow of the CSTR, following dilution, was continuously injected into the ESI source of the TIMS-ToF instrument. The ESI needle was replaced with a 10-cm-long fused silica capillary (0.19 mm outside diameter, 0.1 mm inside diameter; Postnova Z-FSS-100190). Ions were electrosprayed in positive mode with the following settings: voltage, +4.5 kV; nebulizer pressure, 2.0 bar; drying gas flow, 8 l min^−1^; and source temperature, 200 °C. Ion transfer voltages were: quadrupole ion energy, 5 eV; collision cell in, 70 V; collision energy, 8 eV. The ion transmission was optimized for the mass range of interest (*m*/*z* 200–2,000) by using a transfer time of 110 μs, a collision radiofrequency of of 2,000 V peak-to-peak and a prepulse storage time of 10 μs prior to ToF analysis. The mass range scanned by the ToF analyser was *m*/*z* 200–2,000. The instrument was calibrated quadratically using three selected ions from an Agilent ESI liquid chromatography/mass spectrometry tuning mix: (118.0863, 0.542 V·s cm^−2^), (322.0481, 0.732 V·.s cm^−2^) and (622.0290, 0.985 V·s cm^−2^).

### Reservoir computing pipeline

We use a general reservoir computing approach to perform several computational tasks. In all these tasks, we want to approximate a function *f* over a space of inputs $u\in {U}$. Here, the function *f* can represent a binary classification or a smooth function (often constrained to the interval $[\mathrm{0,1}]$). The input space can be one-dimensional or multidimensional, and is normalized to the interval $[\mathrm{0,1}]$ as well.

In all tasks, for every input *u*_*i*_ we assign a true output *y*_*i*_ = *f*(*u*_*i*_). We then use the reservoir to approximate the true outputs as follows. For every input *u*_*i*_ we obtain an enzyme reservoir response *x*_*i*_, consisting of the observed ion signals at steady state as described in the previous sections. This reservoir response can then be transformed into a computational output by multiplying with a weight vector *W* to obtain a prediction ${\hat{y}}_{i}=W{x}_{i}$. By training this weight vector on a (training) set of input/output combinations $\left\{{x}_{i}({u}_{i}),\,{y}_{i}({u}_{i})\right\}$, we can ensure that the reservoir output can approximate the true output.

Depending on the nature of the task, either a linear support vector classifier (for classifications) or a ridge regression (for continuous tasks) is chosen to obtain the weight vector *W*. Both of these training algorithms implement regularization techniques to promote sparser sets of weights, reduce variability in the predicted output and prevent overfitting induced by the high dimensionality of the reservoir output *x*.

A Jupyter notebook with an example pipeline is provided (notebooks/0-classification-example.ipynb).

### Non-linear classification tasks

Three different classification tasks were performed, and four different classification datasets were collected (S1 versus S2, S2 versus *T*, S2 versus pH, and pH versus *T*). For every input in the classification datasets, reactor output was measured for at least 1 h to ensure equilibration of the reservoir response. The output in the last 10 min of this period was averaged to reduce noise. In total 103 features (peptide fragments) were tracked for each dataset. The datasets were independently normalized to remove the mean and scaled to unit variance across features. For the non-linear classification tasks, a LSVC from the scikit-learn Python package^35^ was trained to obtain classifications. For every task and every dataset a repeated stratified L5O CV was performed. Here, a dataset is divided into batches of five inputs (12 batches in total), ensuring every batch has representative inputs from both class labels (stratified), after which the LSVC is trained on all except one batch. The remaining batch of five inputs is then used to calculate a test score. This is done for every batch, and repeated 10 times for different batch-divisions, resulting in 10 × 12 = 120 test-accuracies. These test-accuracies are then used to obtain the final average test-accuracies as well as its s.d.

As test score, Matthew’s correlation coefficient, also known as the *Φ* score, was used:

$$\varPhi =\frac{\mathrm{TP}\times \mathrm{TN}-\mathrm{FP}\times \mathrm{FN}}{\sqrt{\left(\mathrm{TP}+\mathrm{FP}\right)\left(\mathrm{TP}+\mathrm{FN}\right)\left(\mathrm{TN}+\mathrm{FP}\right)\left(\mathrm{TN}+\mathrm{FN}\right)}}$$

where TP denotes the number of true positives, TN true negatives, FP false positives and FN false negatives in the test set. This score returns +1 for perfect predictions and −1 for completely wrong predictions. To convert the score to the accuracies reported in the manuscript, the scores were transformed as

$$\varPhi =\left(\varPhi +1\right)/2$$

before calculating the average and standard deviation over all repeats.

A Jupyter notebook reproducing the analysis is provided (notebooks/1-classification-all.ipynb).

### General information-processing tasks

To demonstrate general information processing using the enzymatic reservoir, the reservoir output was collected at different temperatures, ranging from 25.5 °C to 55 °C at 1.5 °C intervals (21 inputs in total). Similar to the classification tasks, the reservoir was equilibrated for 1 h at every temperature, and the final output was collected as the average over the last 10 min of the equilibration period. Per temperature input, four repeats were collected across different reactors and different days, resulting in a dataset of 82 inputs. Features with a signal-to-noise ratio below 1 (as determined by comparing for every feature, the s.d. in the total dataset to the average s.d. per temperature) were subsequently removed, resulting in 53 significant features in the dataset. The dataset was normalized to remove the mean and scaled to unit variance across features. Different from the classification tasks, a ridge regressor from the scikit-learn Python package^35^ was trained to obtain predictions.

The temperature-sensing task was assessed using a normal repeated L5O-CV (non-stratified) with 100 repeats, and the sensor error was obtained as the average s.d. between the predicted and true temperature for every test set. The binary classification task was assessed similarly to the non-linear classification tasks, but now with 100 repeats. The smooth tasks (activation and tuning) were assessed using a normal repeated L5O-CV (non-stratified) with 100 repeats. Here, the final score was obtained as the *R*^2^ calculated over all repeated L5O-CV test set predictions, where +1 indicates perfect predictions and 0 indicates predictions comparable to a mean estimator.

For the activation, tuning and binary classification task, the task itself depends on the activation/tuning/threshold temperature *T*_0_, defined as:

$${y}_{\mathrm{activation}}=\frac{1}{1+{e}^{-k\left(T-{T}_{0}\right)}}$$

$${y}_{\mathrm{tuning}}=\frac{4}{1+{e}^{-k\left(T-{T}_{0}\right)}}\times \left(1-\frac{1}{1+{e}^{-k\left(T-{T}_{0}\right)}}\right)$$

$${y}_{\mathrm{threshold}}=\left\{\begin{array}{l}1,\,T\ge {T}_{0}\\ \,0,\,T < {T}_{0}\,\end{array}\right.$$

The final scores of the reservoir for each task were calculated for *T*_0_s between 30.75 °C and 51 °C at 1.5 °C intervals, and with *k* determining the sharpness of the activation and tuning tasks. The tuning task has been multiplied by a factor 4 to represent a maximum output of 1 instead of 0.25.

A Jupyter notebook reproducing the analysis is provided (notebooks/2-temperature-processing.ipynb).

### Light-switch tasks

The experiment was conducted at a constant flow rate of 800 µl h^−1^, with a fixed peptide input and the temperature maintained at 30 °C. The reactor was first allowed to reach steady state by running the system for 1 h without any light exposure. Following this, 17 different light-pulse periodicities (from 30 s to 600 s) were applied, keeping a constant cumulative light exposure. Per periodicity, two repeats were collected over a total of 30 min (15 min per repeat) to obtain duplicate measurements. After each pulse, the flow experiment was continued without light exposure for an additional 30 min to allow the system to return to its starting state before proceeding with the next pulse condition. The reservoir output was analysed using HPLC (Supplementary Information, section 3.1.2). The binary classification task was assessed similar to the non-linear classification tasks and the temperature decision-making tasks by repeated stratified L5O-CV with 100 repeats and calculating the average *Φ* accuracy.

A Jupyter notebook reproducing the analysis is provided (notebooks/3-light_sensor.ipynb).

## Online content

Any methods, additional references, Nature Portfolio reporting summaries, source data, extended data, supplementary information, acknowledgements, peer review information; details of author contributions and competing interests; and statements of data and code availability are available at 10.1038/s41557-025-01981-y.

## Supplementary information

Supplementary Information Supplementary Figs. 1–17, Discussion and Tables 1–5.

## Source data

Source Data Fig. 1 HPLC chromatograms corresponding to Fig. 1e.

Source Data Fig. 2 Steady state output data from ESI-MS.

Source Data Fig. 3 Steady state output data from ESI-MS.

Source Data Fig. 4 Peak integral data from HPLC chromatograms.

## Data Availability

The data supporting the findings of this study are available within the article and its Supplementary Information. Processed data are available via Github at 10.5281/zenodo.14906651 (ref. ^36^). Source data are provided with this paper.

## Code availability

Jupyter notebooks for working with the datasets as described in Methods, allowing reproduction of all reservoir computation and reservoir sensing analyses, are provided via Github at 10.5281/zenodo.14906651 (ref. ^36^).
