# Supplementary material for: A recursive enzymatic competition network capable of multitask molecular information processing
Source: Nat Chem. 2025 Oct 17;18(2):302–8. doi: 10.1038/s41557-025-01981-y (PMC12872465; doi:10.1038/s41557-025-01981-y)
Supplement: Supplementary file 1 — Supplementary Figs. 1–17, Discussion and Tables 1–5. [file 41557_2025_1981_MOESM1_ESM.pdf]

nature chemistry

Supplementary information https://doi.org/10.1038/s41557-025-01981-y

A recursive enzymatic competition network capable of multitask molecular information processing

In the format provided by the authors and unedited

Contents

1. Materials and instrumentation 2
1.1. Materials 2
1.1.1. Enzymes 2
1.1.2. Substrates for enzymatic assays 2
1.2. Instrumentation 2
1.2.1. High-performance liquid chromatography 2
1.2.2. Spectroscopy 3
1.2.3. Mass Spectrometry using Compact ESI_TOF MS 3
2. Bead characterization and enzyme assays 4
2.1. Enzyme beads activity assays 4
2.2. Bead stability characterisation 4
3. Experimental methods 4
3.1. Experimental set-up for CSTR containing beads 4
3.1.1. Flow experiments 4
3.1.2. Offline measurements (HPLC) 4
3.1.3. Online measurements (MS) 5
3.2. Characterization of the network 5
3.2.1. Determination of non-linear concentration range for slow substrates 5
3.2.2. Validation of non-linearity within the network 5
3.2.3. Effect of varying pH on network output 5
3.2.4. Effect of varying temperature on network output 6
3.2.5. Dynamic input test 6
3.2.6. Modulation of reservoir complexity 6
3.3. Effect of the photoacid on the network 7
3.3.1. Effect of blue light irradiation on the network output 7
4. Supplementary tables and figures 7
5. References 23

1. Materials and instrumentation

1.1. Materials

1.1.1. Enzymes

a. Trypsin (Tr) from bovine pancreas (Product no. T8003), Type I, solid, ~10,000 BAEE units/mg protein. Purity: 90 – 100 % protein. Mw = 23.8 kDa
b. α-Chymotrypsin (Cr) from bovine pancreas (Product no. C4129), Type II, lyophilized powder, ≥40 units/mg protein. Purity ≥ 85 % protein, Mw = 25 kDa
c. Thrombin (Thr) bovine plasm (Product no. T4648), lyophilized powder, 40-300 NIH units/mg protein (biuret). Purity: 40 – 60 % protein. Mw = 33 kDa (heavy chain), 5 kDa (light chain).
d. Thermolysin (Tln) from Geobacillus stearothermophilus (Product no. T7902), BioReagent, powder, 30-350 units/mg protein (E1%/280). Purity: 20 – 70 % protein. Mw = 34.6 kDa.
e. Elastase (Els), Porcine Pancreas (Product no. 324682), High Purity, lyophilized powder, 250 U/pack, Mw = 25.9 kDa
f. Proline specific Endopeptidase (PEP) from Flavobacterium spp. (Product no. E1411), lyophilized powder, ≥5.0 units/mg solid Mw = 78 kDa
g. Alkaline phosphatase (Alp), from porcine kidney (Product no. P4439), lyophilized powder, ≥100 DEA units/mg protein. Purity = ≥ 50 % protein. Mw = 160 kDa (dimer)

All enzymes are assumed to be salt-free for calculating enzyme concentration, and calculations are based on molecular weights specified above.

1.1.2. Substrates for enzymatic assays

Substrates that were used to run enzyme activity assays are listed alongside the corresponding enzyme in Table S2. Fluorogenic substrates containing cleavable 7-amino-4-methylcoumarin (AMC) were stored as 150 mM stock solutions in anhydrous DMSO at -20 ºC. P-nitrophenolphosphate was stored as a 5 mM stock solution in miliQ at -20 ºC.

1.2. Instrumentation

1.2.1. High-performance liquid chromatography

All HPLC measurements were performed using a Nexera X2 HPLC system with an Inertsil ODS-4 column (3µm, 150×4.6 mm; GLScience) and a guard column (3 µm; 10×4.6 mm) at 40 °C. The elution gradient was as follows: 90% eluent A (MQ with 0.1% v/v TFA), 10 % eluent B (acetonitrile with 0.1 % v/v TFA) for 1min; 90-10 % linear gradient of eluent A for 21 min; 10 % eluent A, 90 % eluent B for 5 min; and 90 % eluent A for 13 min, giving a total runtime of 40 min per measurement. The flow rate was maintained at 0.8 ml/min.

1.2.2. Spectroscopy

Enzymatic assays were performed in a Tecan Spark 10M plate reader. The fluorescence intensity or absorbance of wells containing 60-200 µL of the reaction mixture was monitored for 10-15 min (shaking 3s/orbital mode/amplitude 4mm) at 23 °C using top or bottom reading mode. For enzymes which cleaved AMC-based substrates, $\lambda_{ex}/\lambda_{em}$ =380 nm/460 nm was used. For Thermolysin, the substrate AAGLAN was used which contains a quenched 2-amino benzoyl group. Upon cleavage, the fluorescence increases at $\lambda_{ex}/\lambda_{em}$ = 340 nm/415 nm. The activity of Alkaline Phosphatase was determined using p-nitrophenolphosphate, which, upon removal of the phosphate group, increases absorbance at λ_{max} = 405 nm.

For flow experiments where the output was measured online using fluorescence, an Avantes DUO spectrofluoromiter connected to an Avalight 355 nm LED lamp was used. The fluorescence of the reactor output (λem = 450-460 nm, integration time = 250 ms) was measured through PTFE tubing (0.25 mm inner diameter) using a custom made tubing holder.

1.2.3. Mass Spectrometry using Compact ESI_TOF MS

For dynamic input reactions, 10 uL of the reactor output at each sample timestep was directly injected via a Vanquish UHPLC (ThermoFisher Scientific) autosampler into the electrospray ionisation (ESI) source of an Compact QTOF mass spectrometer (Bruker Daltonics, Germany). The ESI needle was replaced with a 10 cm long fused silica capillary (0.19 mm outside diameter, 0.1 mm inside diameter– Postnova Z-FSS-100190). Ions were electrosprayed in positive mode with the following settings: Voltage +4.5 kV, nebulizer pressure 2.0 Bar, drying gas flow 6 Lmin-1, and source temperature set to 200 °C. Ion transfer voltages were Quadrupole Ion Energy = 5 eV, Collision Cell In = 300 V and Collision Energy = 8 eV. The ion transmission was optimized for the mass range of interest (m/z 200-2000) by using a transfer time of 110 µs, a collision RF of 2250 Vpp and a pre pulse storage time of 5 µs prior to time-of-flight (ToF) analysis. The mass range scanned by the ToF analyser was m/z 200-2000. The MS dimensions were calibrated quadratically using three selected ions from the Agilent ESI LC/MS tuning mix [(118.0863), (322.0481), and (622.0290)].

2. Bead characterization and enzyme assays

2.1. Enzyme beads activity assays

For assays, the corresponding reporting substrate (500 µM, list in Table S2) was combined with enzyme beads (4 µL) in phosphate buffer (50 mM, pH 7,4) in a 384 well Greiner flat bottom, transparent polypropylene plate (Greiner, Item No.: 781201)**.** This was then placed in a Tecan Spark M10 plate reader and the reaction was followed by either fluorescence (for AMC containing substrates and AAGLAN) or absorbance (for p-nitrophenyl phosphate) over 15 minutes. A calibration graph was made by measuring the fluorescence and absorbance of varying concentrations of AMC and p-nitrophenol respectively. For Thermolysin, the enzyme was allowed to react completely with a known concentration of AAGLAN allowing calculation of the product concentration at each timestep. The results of the measurement are plotted in Figure S1.

2.2. Bead stability characterisation

Empty gel beads, prepared following the procedure described in Baltussen et al,[1] were imaged using an IX83 microscope. The average diameter was 70 ±3 µM.

The beads were then heated to 60 ºC for 1 hour, as can be seen in Figure S2, the beads remained stable and showed no structural changes.

3. Experimental methods

3.1. Experimental set-up for CSTR containing beads

3.1.1. Flow experiments

Experiments were generally conducted in a fluidic set-up with flowrates and temperature controlled using a LabM8 (Labm8 - Open Source Microfluidics) pump unit. Flowrates were input to the LabM8 Web Control as a series of movement steps consisting of 15 - 40 minutes which were sent line by line to the stepper motor which controlled each syringe pump giving an overall flowrate of 400 µL/h. The temperature of the aluminium plate comprising the base of the CSTR was also controlled by the same central unit and was input as lines of code between movement steps using LabM8 Web Control.

3.1.2. Offline measurements (HPLC)

Where the output was measured offline using high-performance liquid chromatography, the CSTR output would be collected using an autosampler set to rotate sample collection vessel every 15 minutes. Once collected, the output was transferred to sample vials and measured on a Shimadzu Nexera X2 HPLC system, using injection (50 µL) and measurement parameters as described above.

3.1.3. Online measurements (MS)

Where the output was measured online, the output of the reactor would flow to the inlet of a timsTOF mass spectrometer (fig. S3). The CSTR outlet was connected first to an Inline-checkvalve (Check Valve Inlet Assembly 3 psi 1/4-28 .020, Part# CV-3315, IDEX) to prevent backflow prior to dilution with MQ using a Bruker Elute HPG 1300 high-performance liquid chromatography system at a flowrate of 0.046 $mLmin^{-1}$. Pressure was maintained at 2 bar within the system using a Restek RT-25020 backpressure regulator connected to a waste line . The dilution and backpressure regulators were sequentially connected between the reactor outlet and the MS inlet using Y-connectors.

3.2. Characterization of the network

3.2.1. Determination of non-linear concentration range for slow substrates

A dynamic concentration range was determined for the slow substrates, CCFSWRCRC (chymotrypsin, Cr) and IYPFVEPI (proline specific endopeptidase, PEP). For this, online spectroscopic measurements were performed to measure the fluorescence intensity of the reactor output where concentrations of the slow peptide substrates were varied from 10-100 µM, while keeping the concentration of the reporting substrate (Suc-AAPF-AMC and Z-GP-AMC for Cr and PEP respectively) constant (100 µM). The concentration of AMC at steady state was calculated and plotted against the slow substrate concentration in Figure S4. This provides a range within which the concentration of slow substrate has a non-linear effect on the activity of the respective enzymes. This range was determined to be between 15-75 µM.

3.2.2. Validation of non-linearity within the network

To ensure that the transformation of the peptide input is suitably non-linear, each peptide was individually run through the reactor with all enzyme beads at maximum experimental concentrations (100 µM for peptides **1** and **2**, 75 µM for peptides **3**–**5**, and 160 µM for peptides **6** and **7**). In each case, reactions were allowed to reach steady-state, and outputs were measured via HPLC, normalized, and combined. Subsequently, a mixture of all peptides at the same concentrations was simultaneously flowed through the reactor, and the steady-state output was measured using the same method. The results are shown in Figure 1d.

3.2.3. Effect of varying pH on network output

Cleavage fingerprints for the network were analysed using the protocol described in section 3.2.2 under three pH conditions (6, 7, and 8) at a constant temperature of 30°C (Cleavage fingerprint: individual peptide, fig. S5 and peptide mixture, fig. S6).

3.2.4. Effect of varying temperature on network output

To check the effect of temperature on the network, cleavage fingerprints were analysed using the protocol described in section 3.2.2 under three different temperatures (30, 40, and 50°C) at a constant pH of 7.4 (Cleavage fingerprints: individual peptide, fig. S7 and peptide mixture, fig. S8).

3.2.5. Dynamic input test

A reactor was charged with 4 uL each of 6 enzymes from the protease network (trypsin, chymotrypsin, thrombin, thermolysin, elastase and prolyl endopeptidase) immobilised on hydrogel beads. A syringe was filled with 6 peptides from the network at concentrations determined to be the maximum of each peptides dynamic range: AVNIPFKVHLRCKAAFC and SSVRWWSDDEWRW (100 µM), IYPFVEPI and CCFSWRCRC (75 µM), and TKIFKI and TTMHPRL (160 µM). A varying input was generated from two syringes, one containing the peptide mixture as described and one containing only buffer, such that the overall flow rate remained constant and the ratio of the inputs was equal to the y value of a sin wave with 25 min periodicity and an amplitude of 0.5. A sine wave with 25 minute periodicity and an amplitude of 1 was created by adjusting the ratio of the flow rates of the two syringes. The output was sampled every 4.5 minutes for approximately 7 hours. The outputs were measured using a BRUKER Compact ESI-TOF mass spectrometer. 103 ion traces were extracted and a ridge regressor was trained to predict the input at 9 and 13.5 minutes ahead. The results of the prediction are shown in figure S12 a-e alongside the RMSE for each timestep.

3.2.6. Modulation of reservoir complexity

To explore how computational capacity scales with system complexity, we performed experiments to check how the reservoir performs when it consists of 3 (chymotrypsin, thrombin, thermolysin) prolyl and 5 (chymotrypsin, thrombin, thermolysin, prolyl endopeptidase, alkaline phosphatase) enzymes compared to the full 7 enzymes described in the main manuscript. We used the same chemical input profile (fig. 2c) and performed the same classification tasks ('XOR', 'Circle' and 'Hourglass') as shown in Figure 2b. Reducing the number of enzymes led to fewer peptide cleavage events, thereby minimizing the nonlinear complexity typically achieved with the full network.

We summarized the classification performance using a bar plot in Figure S14, where scores are grouped by classification task and color-coded based on the number of enzymes used in the reservoir. In both the 3- and 5-enzyme cases, the L5O-CV Φ accuracy scores float around 0.5, indicating performance near chance level and suggesting that the reservoir effectively predicts only half of the tasks. In contrast, the 7-enzyme system achieves higher accuracy, demonstrating its effectiveness as a reservoir computer.

3.3. Effect of the photoacid on the network

Synthesis and characterisation of the photoacid is reported by Wimberger et al.[2] HPLC chromatogram of the photoacid is shown in Figure S10. Spectral data matches those reported in the literature.

3.3.1. Effect of blue light irradiation on the network output

Blue light irradiation induces the transition of merocyanine (open form) to spiropyran (closed form), releasing a proton and lowering the pH.[2] To test this pH-lowering effect on network output, peptides were dissolved at previously mentioned concentrations in 5 mM phosphate buffer containing 7 mM merocyanine. The peptide-photoacid mixture was introduced into the reactor containing enzyme beads, and reactions were allowed to reach steady states. The reactor was then irradiated with blue light for one hour, followed by an hour in the dark. Outputs were collected and analysed using HPLC, and chromatograms were compared for light-on and light-off conditions (fig. S11).

4. Supplementary tables and figures

No.	Peptide	M.W. (as reported by CASLO)
1	AVNIPFKVHLR<u>C1</u>KAAF<u>C1</u> (one disulfide bonds: C1-C1)	1915.03
2	SSVRWWSDDEWRW	1795.31
3	Ac-<u>C1C2</u>FSWR<u>C2</u>R<u>C1</u> (two disulfide bonds: C1-C1, C2-C2)	1202.78
4	Ac-<u>C1C2</u>F(pS)WR<u>C2</u>R<u>C1</u> (two disulfide bonds: C1-C1, C2-C2)	1281.47
5	IYPFVEPI	976.64
6	TKIFKI	748.48
7	TTMHPRL	854.64

Table S1: List of commercial peptides used in network.

Enzyme	Fluorogenic Substrate
Tr	Bz-Arg-AMC (Bz-R-AMC) (Bachem, product no. 4002540)

Cr	Suc-Ala-Ala-Pro-Phe-AMC (Suc-AAPF-AMC) (Bachem, product no. 4012873
Thr	Bz-Phe-Val-Arg-AMC (Bz-FVR-AMC) (Bachem, product no. 400313)
Els	MeOSuc-Ala-Ala-Pro-Val-AMC (MeOSuc-AAPV_AMC) (Bachem, product no. 4005227
PEP	Z-Gly-Pro-AMC (Z-GP-AMC) (Bachem, product no. 4002518)
Tln	Abz-Ala-Gly-Leu-Ala-p-nitrobenzylamide (Abz-AGLA-pNBA) (Bachem, product no. 4014232)
Alp	P-nitrophenolphosphate (Sigma-Aldrich, product no. 4876)

Table S2: Substrate used to determine the activity of the corresponding enzyme and product number for each substrate.

**Enzyme**	**Cleavage sites**
Tr	R, K at position P1
Cr	W, Y, F at position P1
Thr	P in position P2, R in position P1
Els	A, V, L at position P1
PEP	P in position P1
Tln	I, L, A, V, M, W in position P1'
Alp	Phosphate group

Peptide schematic: H_2N-P3-P2-P1—P1'-P2'-P3'-COOH (cleavage site P1-P1')

Table S3: Potential cleavage sites for the corresponding enzymes in the network.[3]

**peak No.**	**m/z**
1	259.213
2	262.907
3	281.194
4	284.889
5	296.17
6	300.905
7	301.152
8	323.134
9	345.116
10	361.198
11	383.181
12	395.764
13	401.153
14	415.204
15	425.237
16	426.829
17	435.141

18	436.183
19	439.219
20	447.22
21	459.109
22	461.201
23	470.214
24	481.092
25	482.248
26	497.799
27	498.9
28	508.791
29	510.19
30	513.303
31	517.068
32	519.365
33	519.782
34	524.807
35	533.234
36	535.284
37	541.347
38	546.788
39	550.323
40	557.267
41	566.888
42	568.77
43	572.158
44	572.305
45	590.752
46	594.287
47	608.247
48	617.282
49	617.759
50	626.317
51	628.749
52	630.229
53	639.741
54	644.765
55	644.902
56	648.298
57	650.731
58	663.176
59	666.747
60	668.328
61	670.279
62	674.167
63	688.729
64	699.722
65	710.711
66	730.11
67	730.262
68	732.693
69	759.699
70	764.348
71	766.203
72	770.69
73	780.876
74	781.681
75	808.687
76	812.196
77	830.669
78	834.178
79	838.837
80	841.66
81	844.107
82	852.651
83	855.451
84	866.236
85	867.029
86	874.633
87	909.516
88	912.631
89	923.621
90	928.646
91	950.629
92	960.455
93	961.619
94	972.61
95	980.081
96	994.592
97	999.515
98	1021.498
99	1043.479
100	1043.58
101	1054.571
102	1179.453
103	1201.415

Table S4: List of assigned ion traces m/z values extracted with a mass width of 0.02 Da.

**No. of peaks**	**Retention time range for peak maxima approx. (min)**
1	10.35 ± 0.05
2	10.47 ± 0.05
3	10.9 ± 0.05
4	11.33 ± 0.05
5	11.53 ± 0.05

6	11.78 ± 0.05
7	12.06 ± 0.03
8	12.26 ± 0.03
9	12.44 ± 0.05
10	12.55 ± 0.05
11	12.75 ± 0.05
12	12.85 ± 0.03
13	13.03 ± 0.04
14	13.16 ± 0.05
15	13.4 ± 0.05
16	13.71 ± 0.03
17	13.8 ± 0.03
18	13.93 ± 0.03
19	14.18 ± 0.03
20	14.41 ± 0.03
21	14.7 ± 0.02
22	14.92 ± 0.04
23	15.21 ± 0.03
24	15.4 ± 0.02
25	15.59 ± 0.03
26	15.74 ± 0.03
27	15.86 ± 0.03
28	16.16 ± 0.02
29	16.93 ± 0.03

Table S5: List of observed HPLC peaks with their respective retention time ranges of the peak maxima (used for light pulse experiment)

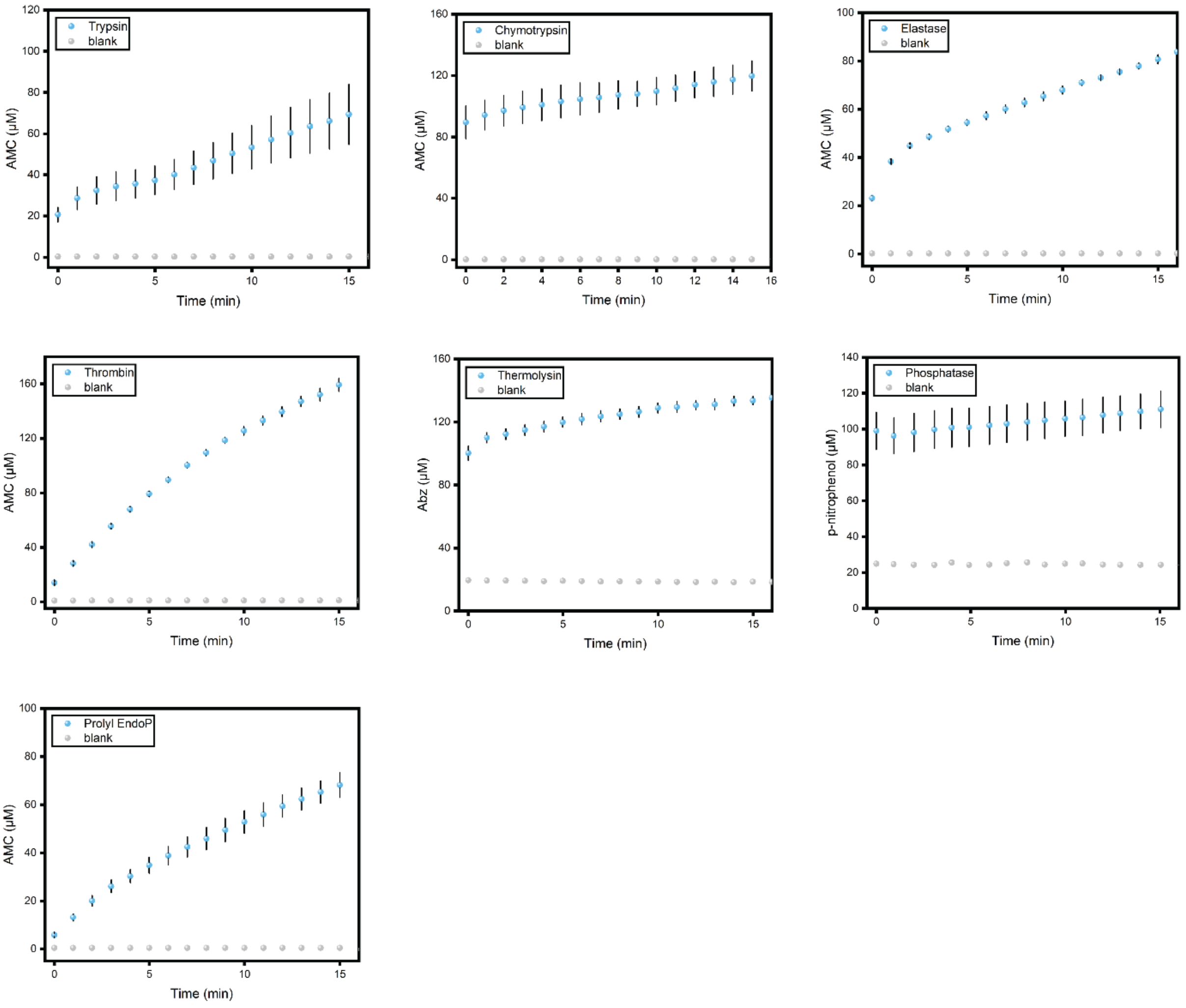

Figure S1: Activity assays (duplicates) for enzyme beads performed using 0.5 mM of substrate, with controls conducted in the absence of enzyme beads. Data are presented as mean values +/- SEM.

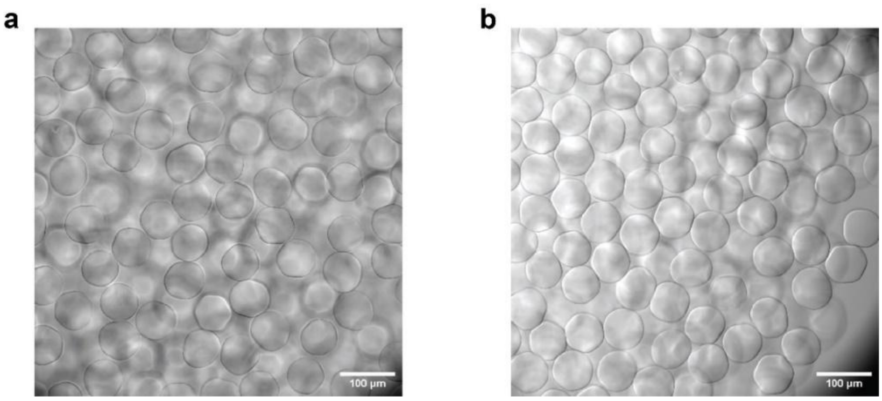

Figure S2: Temperature stability for hydrogel beads, a) before heating; b) after heating for 1-hour at 60˚C.

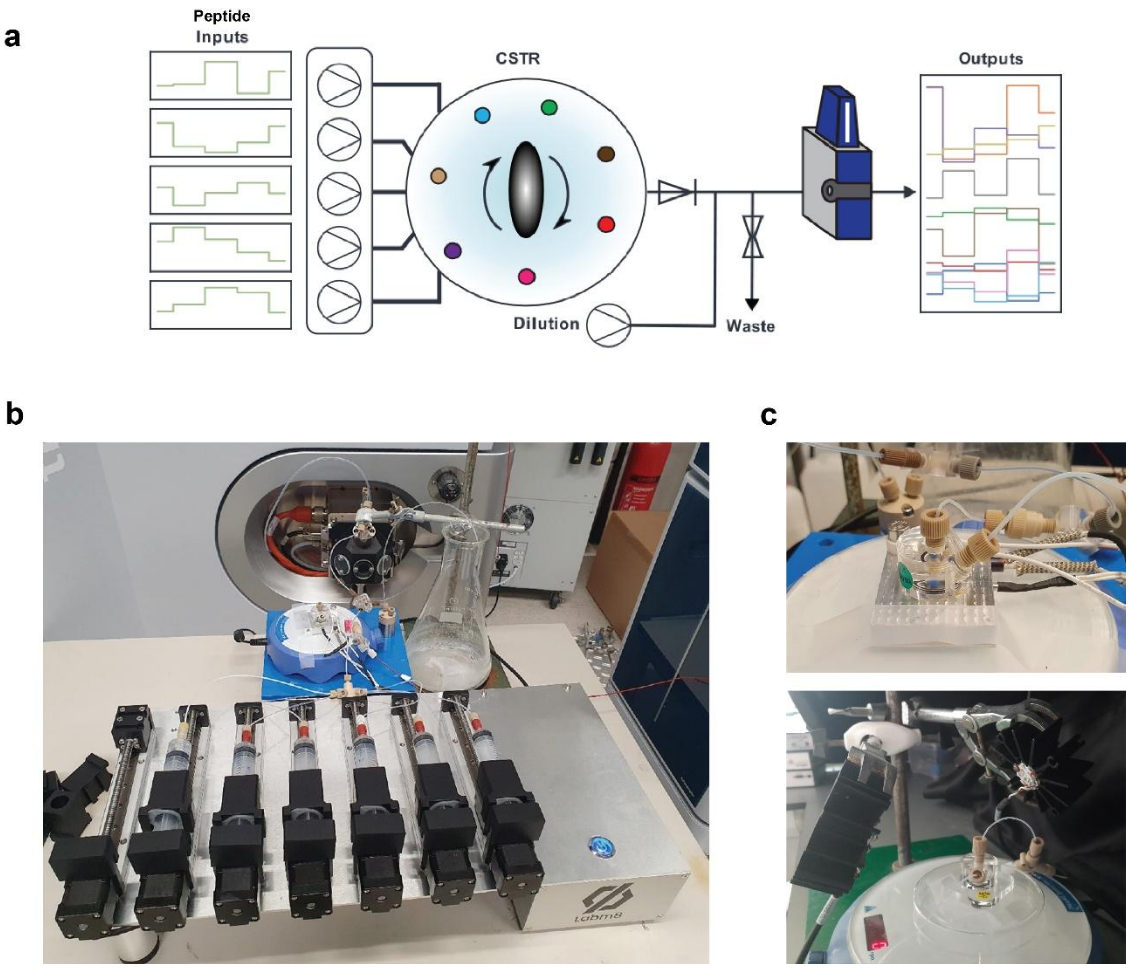

Figure S3: a) Schematic of the experimental setup for experiments conducted in flow where output was measured using MS. b) Microfluidics pumps setup with syringe flowing to the CSTR with effluent flowing to the TimsTOF. c) CSTR with inlet and outlet tubings mounted on temperature stage (top) and LED setup for irradiation (bottom).

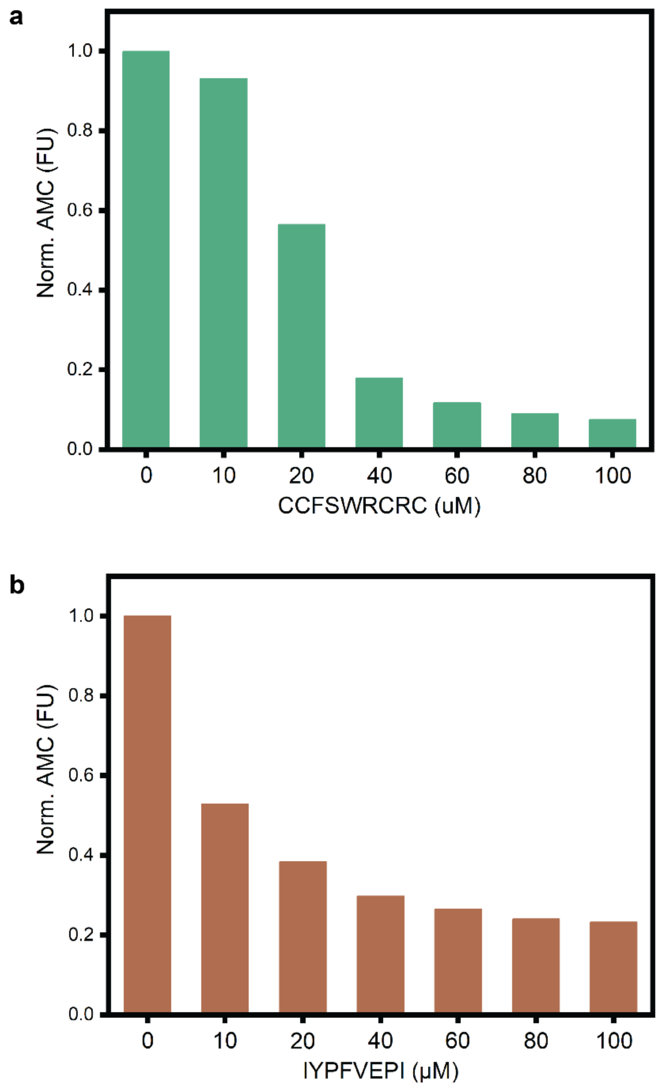

Figure S4: Enzyme inhibition study in the presence of peptide inhibitors (slow substrates): a) effect of CCFSWRCRC on chymotrypsin and b) effect of IYPFVEPI on proline specific endopeptidase.

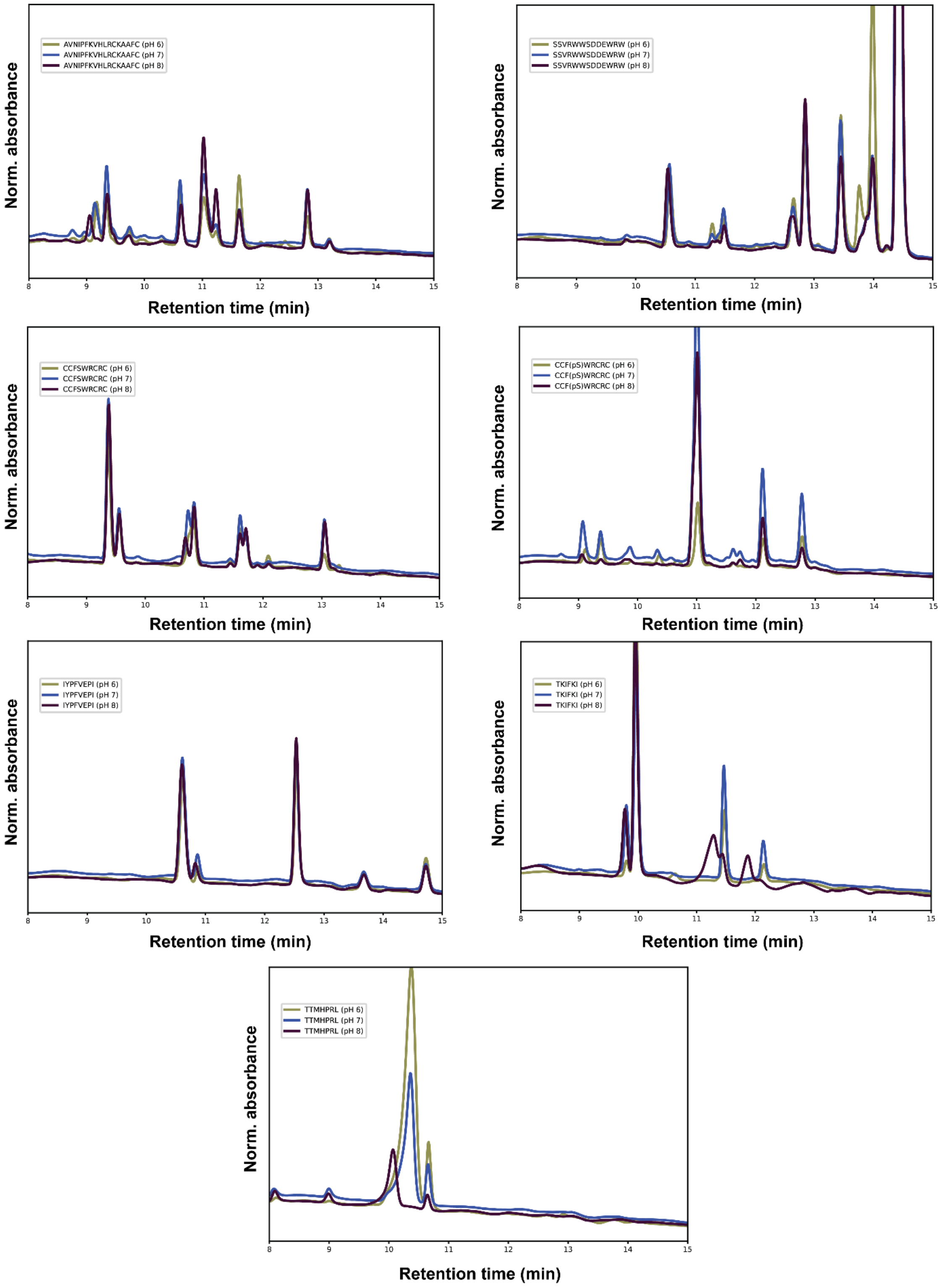

Figure S5: Investigation of cleavage fingerprint of individual peptide (HPLC chromatograms) with varying pH (6, 7, and 8) at room temperature (RT).

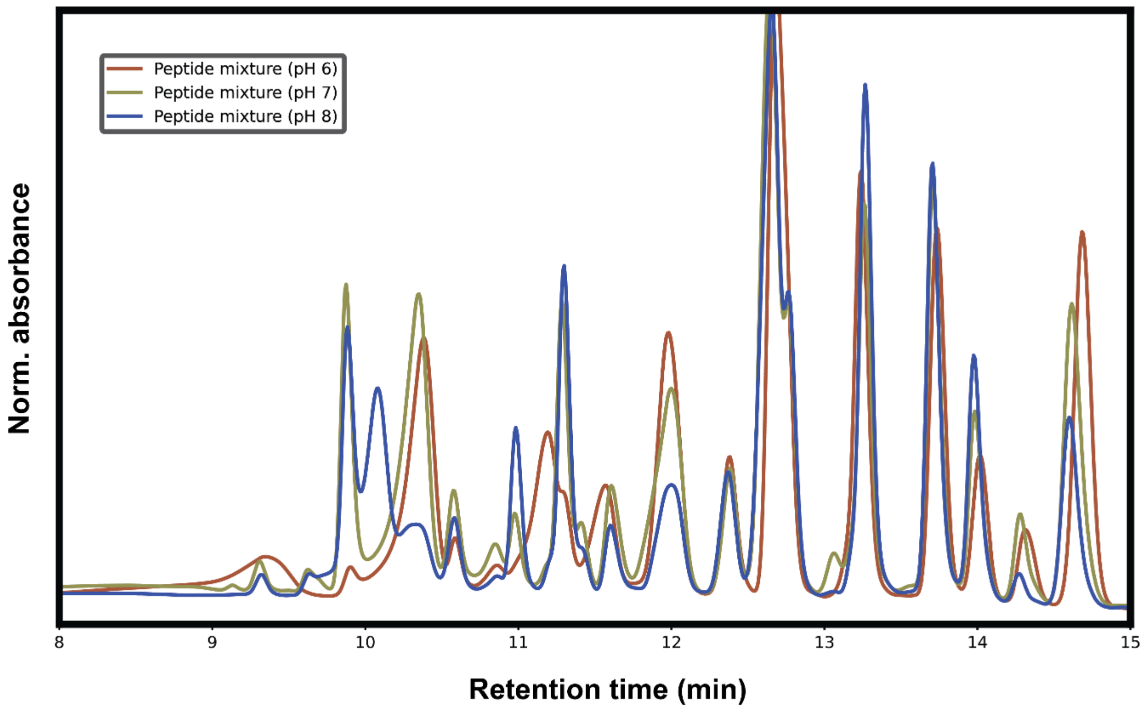

Figure S6: Investigation of cleavage fingerprint of peptides mixture (HPLC chromatograms) with varying pH (6, 7, and 8) at RT.

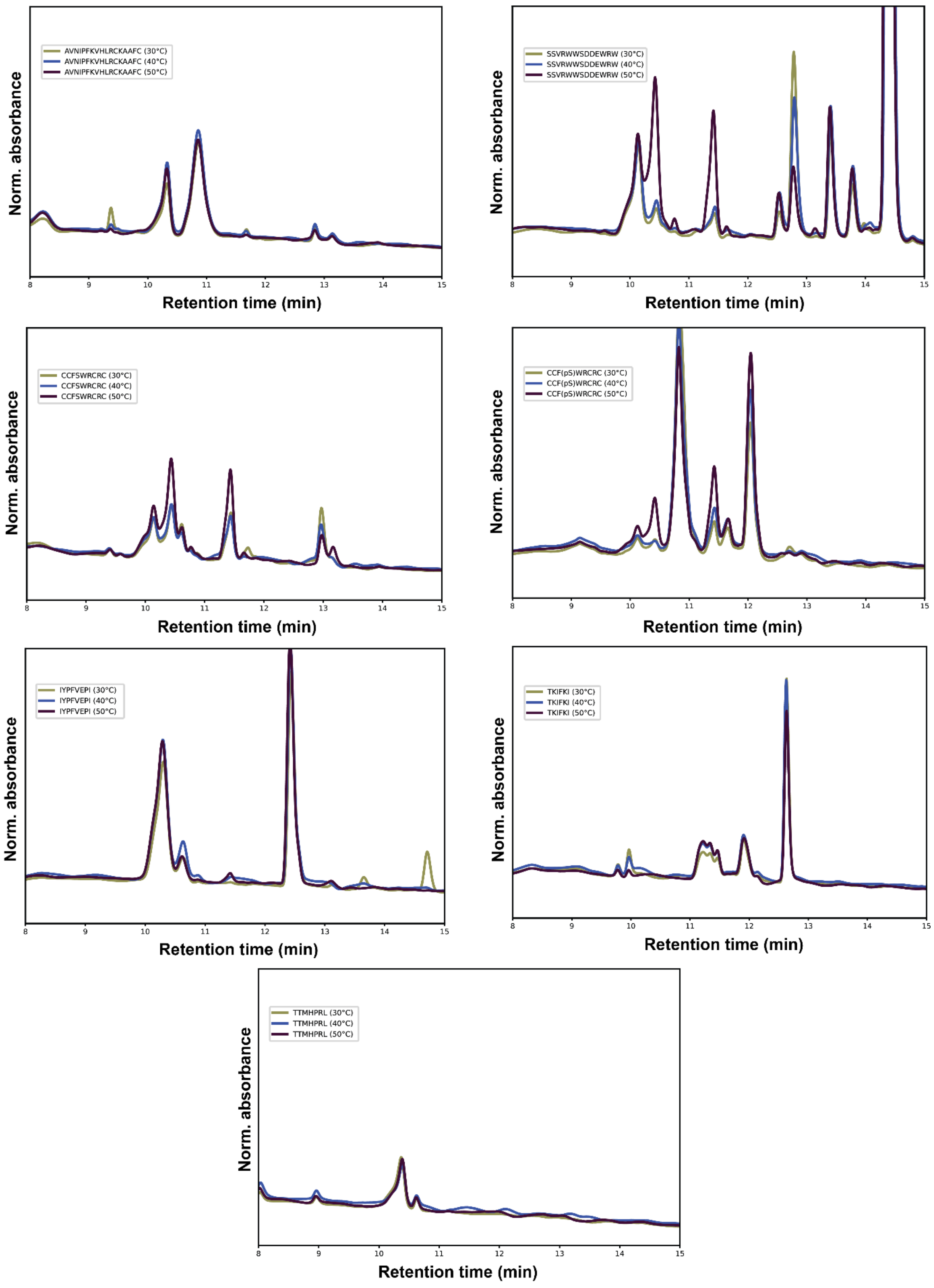

Figure S7: Investigation of cleavage fingerprint of individual peptide (HPLC chromatograms) with varying pH (30˚C, 40 ˚C, and 50 ˚C) at constant pH 7.4.

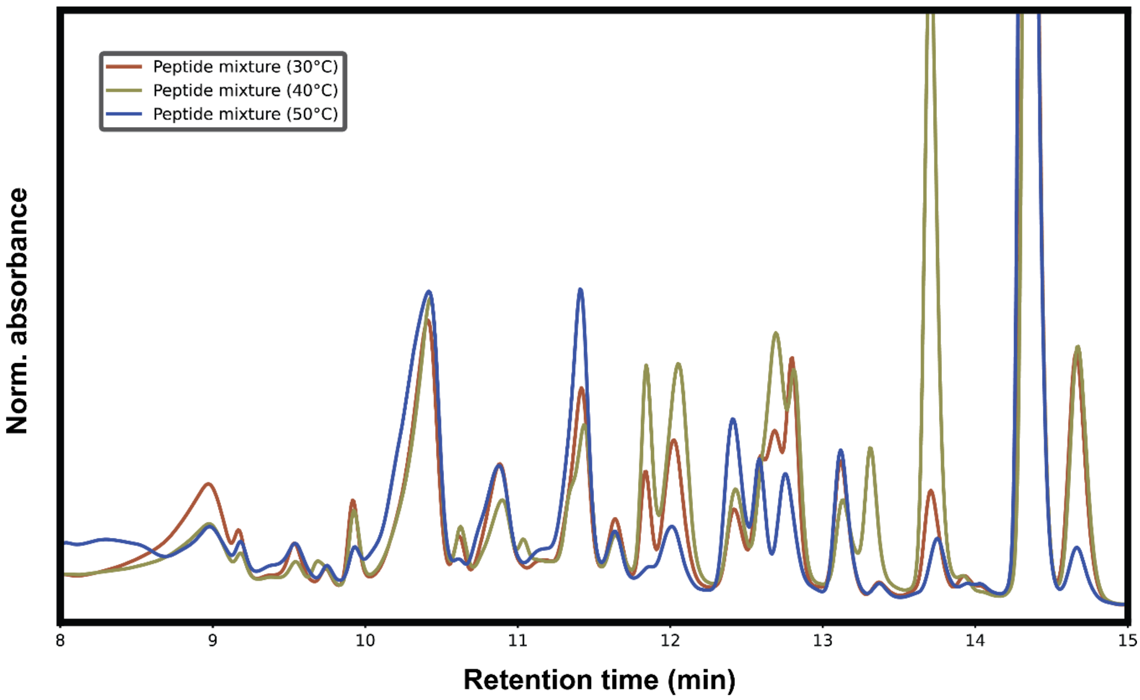

Figure S8: Investigation of cleavage fingerprint (HPLC chromatograms) of peptides mixture with varying pH (30˚C, 40 ˚C, and 50 ˚C) at constant pH 7.4.

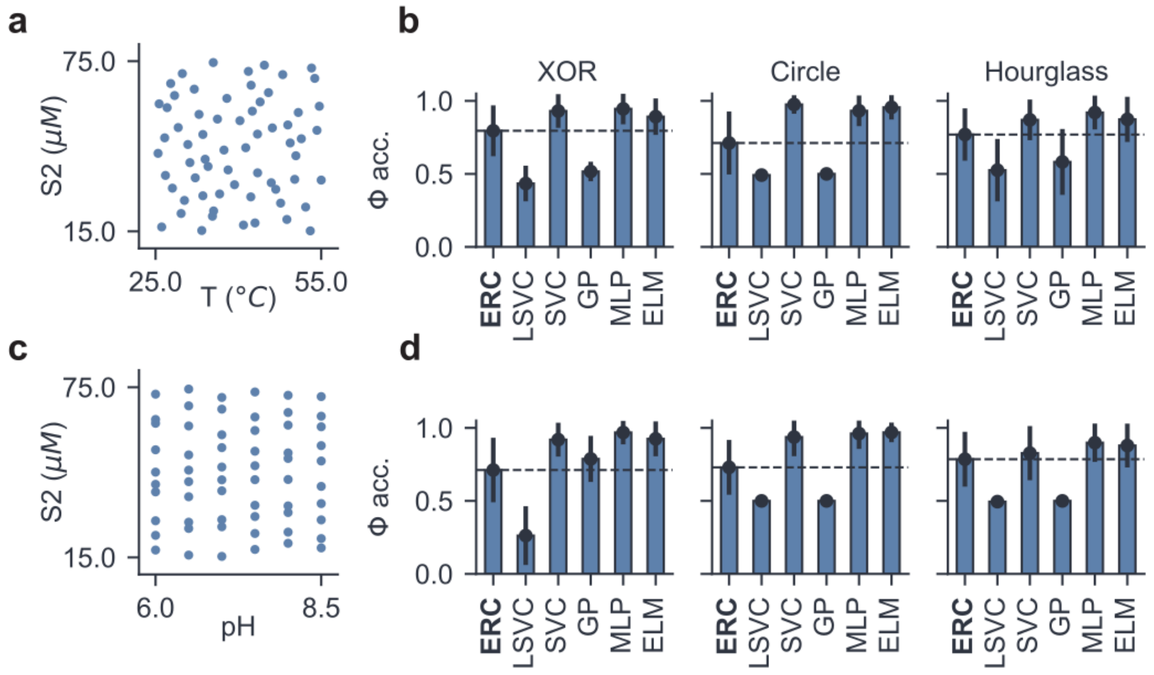

Figure S9: a) Inputs used for the peptides- temperature classification tasks **b)** Mean L5O-CV Φ accuracy over splits and 10 repeats, with error bars indicating standard deviation. Shown tasks correspond to the XOR, circle and hourglass classification for the substrate input space, with the reservoir response (ERC), the training algorithm without reservoir (LSVC), and several machine learning algorithms (SVC – Support Vector Classifier, GP – Gaussian Process, MLP – Multi-layer

Perceptron, ELM – Extreme Learning Machine). **c)** Inputs used for the peptides - pH classification tasks. **d)** Mean L5O-CV Φ accuracy over splits and 10 repeats, with error bars indicating standard deviation. Shown tasks correspond to the XOR, circle and hourglass classification for the physicochemical input space. Abbreviations as in S9b.

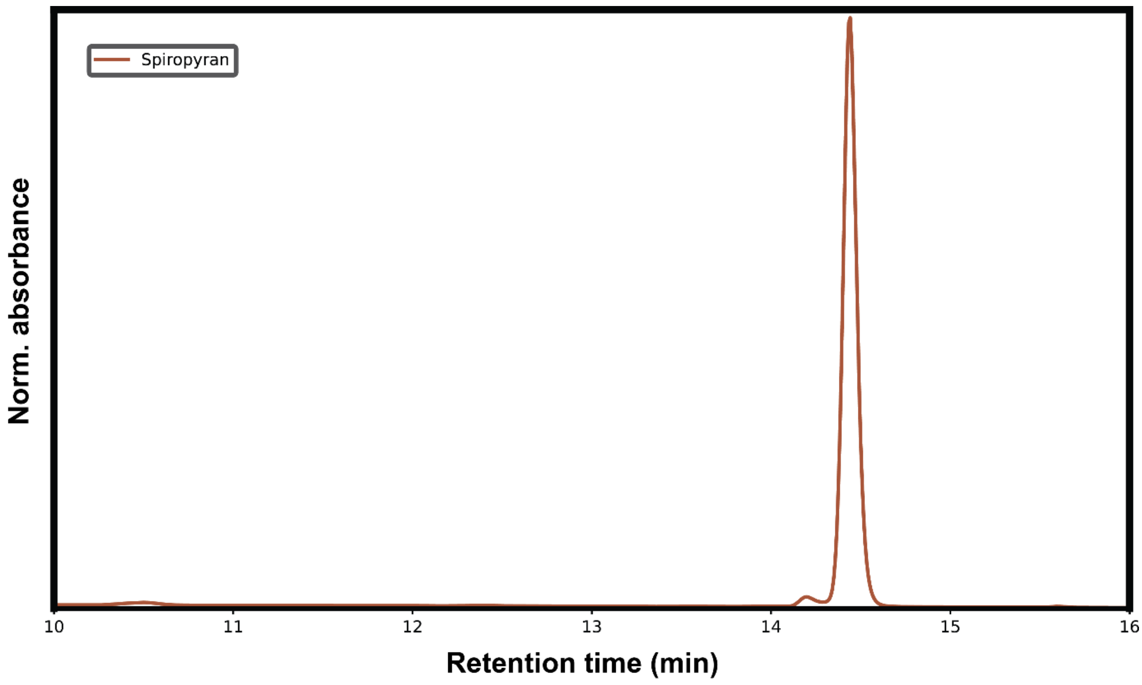

Figure S10: HPLC of the photoacid (7 mM in 50 mM phosphate buffer)

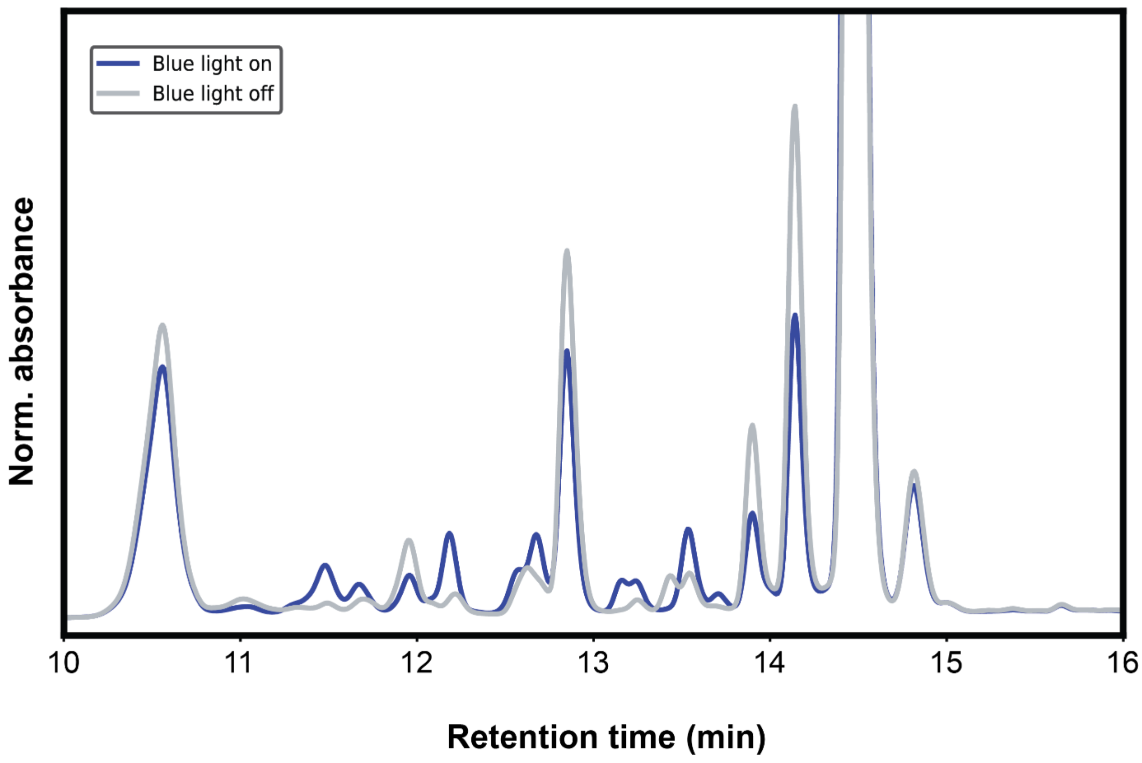

Figure S11: HPLC of the cleavage fingerprints of the network with and without blue light exposure in the presence of photoacid.

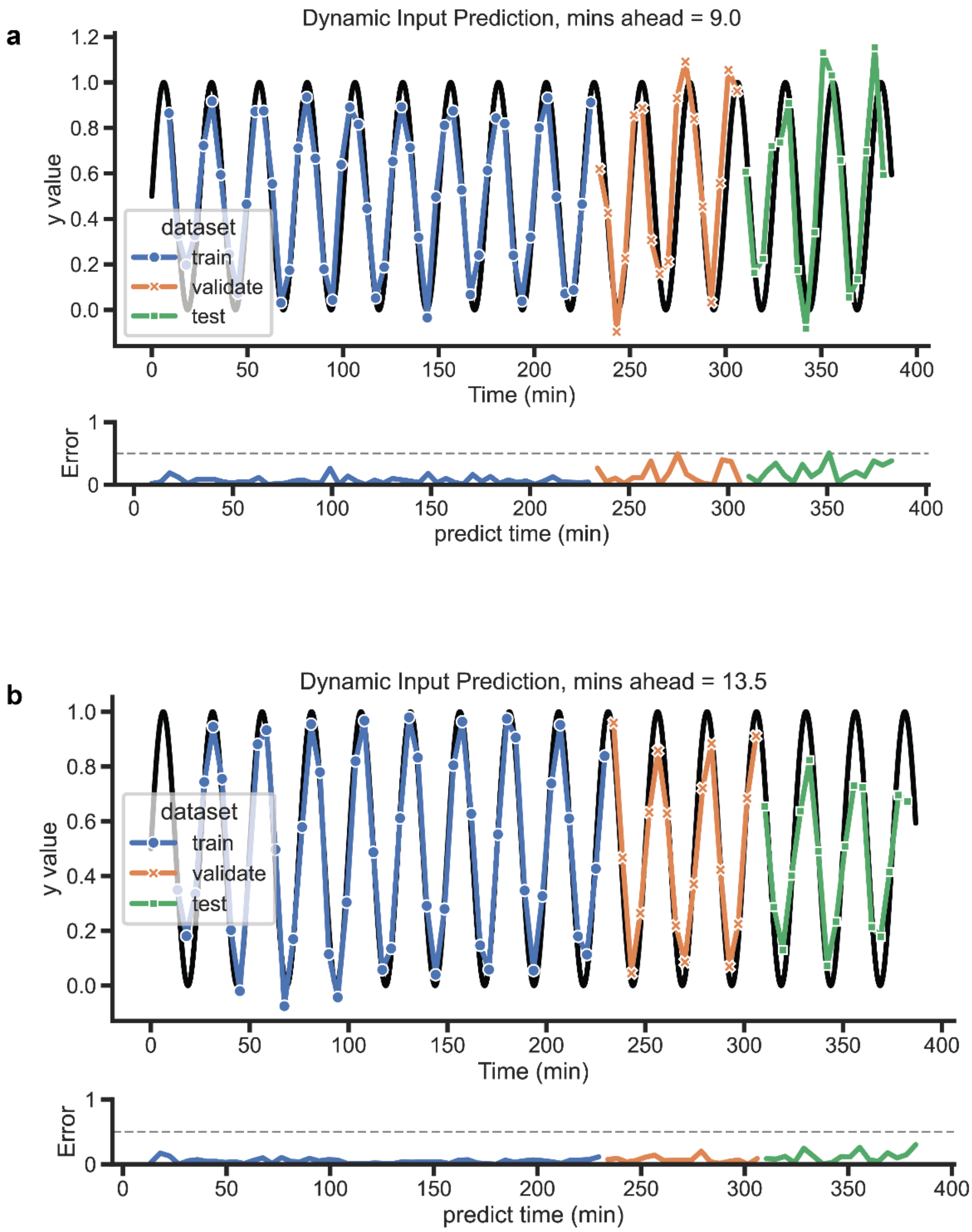

Figure S12: Plots showing the dynamic input prediction capabilities of the ERN. Training (blue), validation (orange) and test (green) predictions for a sin input a) 9 and b) 13.5 minutes in the future.

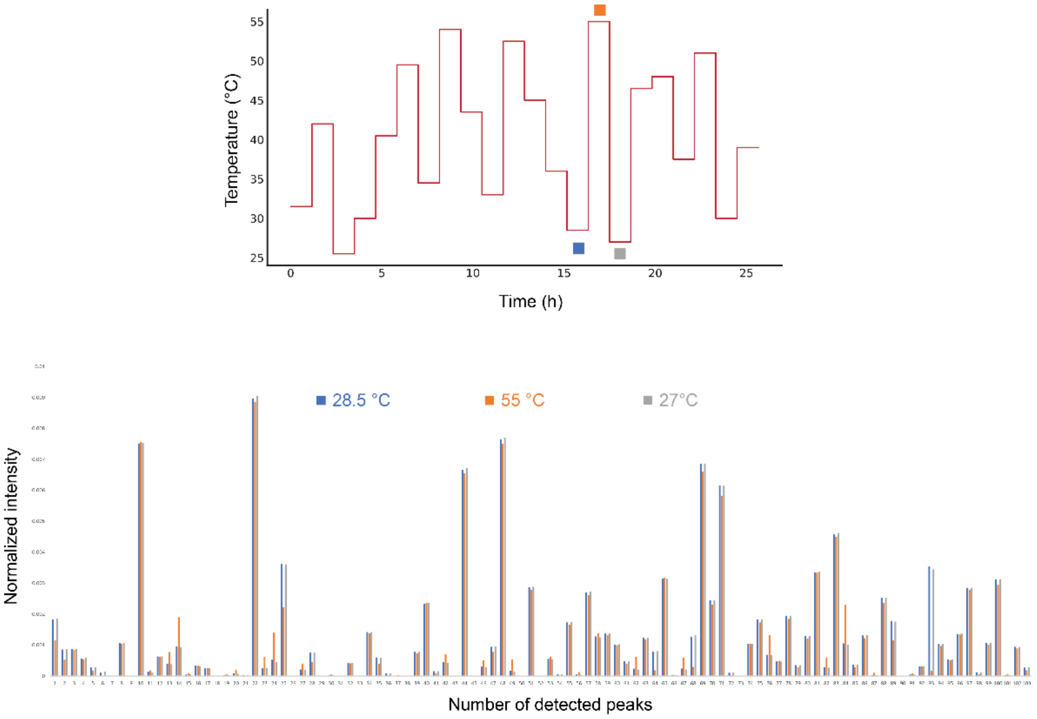

Figure S13: (top) The temperature sequence used for the experiment described in Figure 3. (bottom) Normalized intensity of the reservoir outputs at the steady states for 28.5 °C (blue), 55 °C (orange) and 27 °C (grey), as detected by ESI-MS.

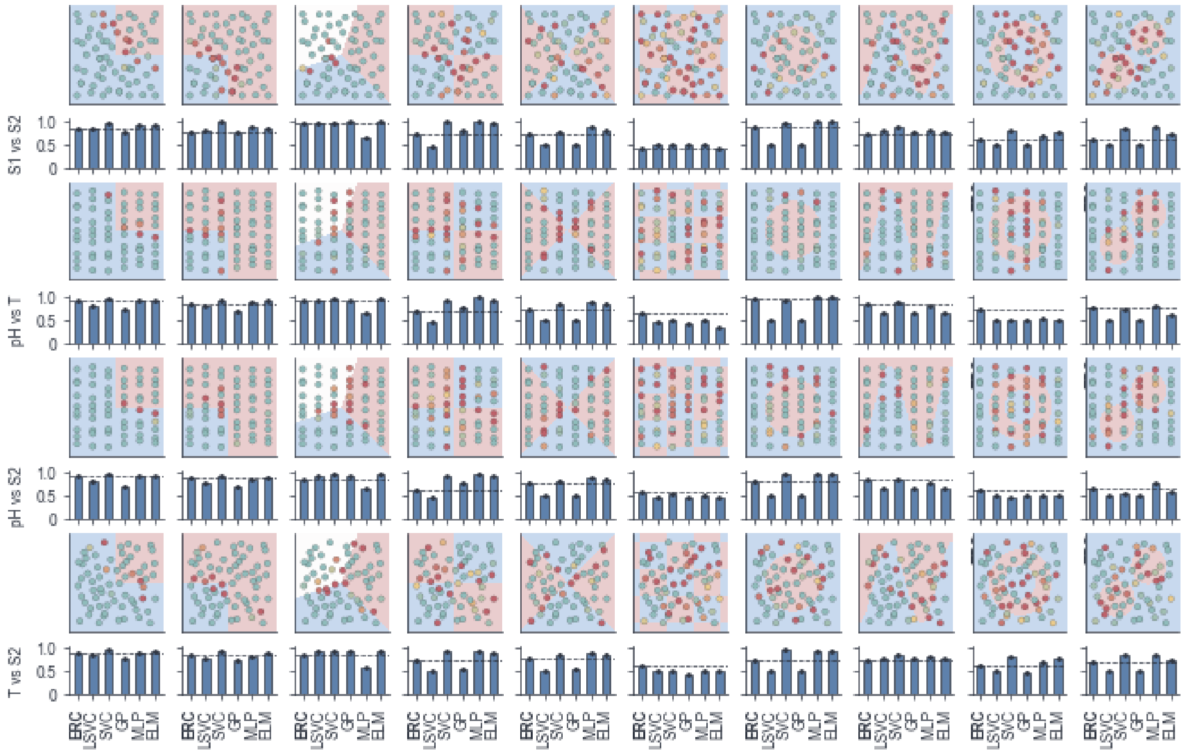

Figure S14: Extended results for reservoir classification tasks (AND, OR, Triangle, XOR, Hourglass, Checkers, Circle, Sine, Eye, and Dots) performed with the enzymatic reservoir computer (ERC) using

(first row) chemical inputs S1 vs S2, (second row) pH vs temperature, (third row) pH vs S2, and (fourth row) temperature vs S2.The bar chart below each classification task shows a comparison between L5O-CV Φ accuracy for the enzymatic reservoir computer (ERC), with various other machine learning classifiers. The dashed line indicates the score achieved by the ERC.

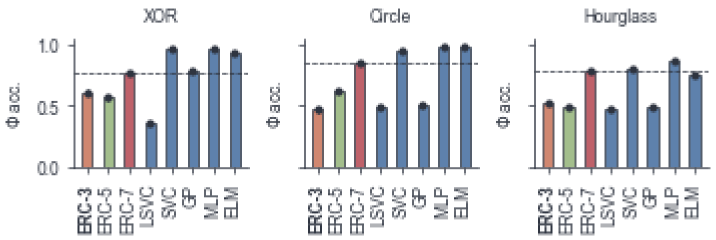

Figure S15: Classification scores for the XOR, Circle, and Hourglass tasks performed using reservoirs composed of 3, 5, and 7 enzymes. All tasks were conducted using the chemical input profile shown in Figure 2c.

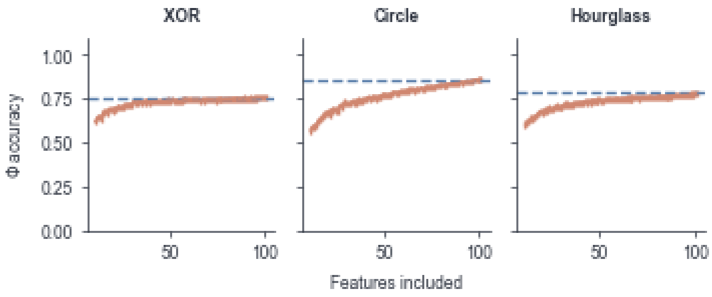

Figure S16: Classification accuracies for the XOR, Circle, and Hourglass tasks performed with varying sizes of the output (feature) space, from 11 to 103 features. All tasks were conducted using the chemical input profile shown in Figure 2c. For every data point, the indicated number of features were randomly selected from the full set of features, and the final accuracy was calculated as the L5O-CV averaged over 100 different selections of the features.

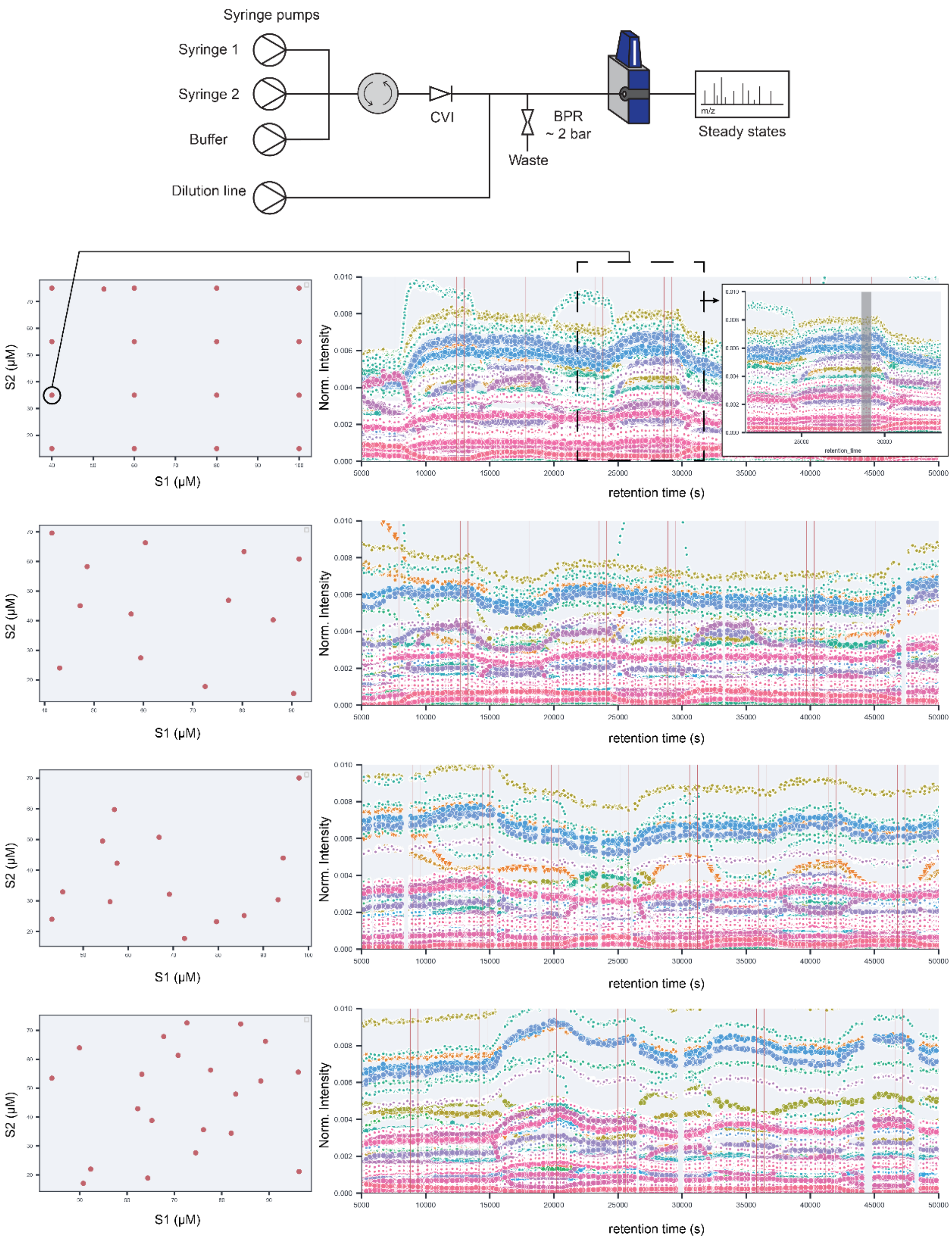

Figure 17: (top) Schematic experimental workflow for the nonlinear classification experiment discussed in Figure 2b. (left) Inputs corresponding to classification tasks performed varying S1 and S2. (right) The reservoir response at the steady states for varying inputs. Areas between vertical bars were averaged and normalised to use as chemical output. The inset in the first time trace zooms in on the response for a specific input condition (circled in the left panel) to showcase the system indeed reaches a steady state. The grey bar represents the region used to extract steady state output for that particular condition.

5. References

1. Baltussen, M. G., van de Wiel, J., Fernández Regueiro, C. L., Jakštaitė, M. & Huck, W. T. S. A Bayesian Approach to Extracting Kinetic Information from Artificial Enzymatic Networks. *Anal. Chem.* **94**, 7311–7318 (2022). 1.
2. Wimberger, L., Andréasson, J. & Beves, J. E. Basic-to-acidic reversible pH switching with a merocyanine photoacid. *Chem. Commun.* **58**, 5610–5613 (2022).
3. Gasteiger, E., Hoogland, C., Gattiker, A., Duvaud, S., Wilkins, M.R., Appel, R.D. & Bairoch A.; Protein Identification and Analysis Tools on the Expasy Server; (In) John M. Walker (ed): The Proteomics Protocols Handbook, *Humana Press* (2005).
